# Tissue-Specific Knockdown of Genes of the *Argonaute* Family Modulates Lifespan and Radioresistance in *Drosophila melanogaster*

**DOI:** 10.3390/ijms22052396

**Published:** 2021-02-27

**Authors:** Ekaterina Proshkina, Elena Yushkova, Liubov Koval, Nadezhda Zemskaya, Evgeniya Shchegoleva, Ilya Solovev, Daria Yakovleva, Natalya Pakshina, Natalia Ulyasheva, Mikhail Shaposhnikov, Alexey Moskalev

**Affiliations:** 1 Laboratory of Geroprotective and Radioprotective Technologies, Institute of Biology, Komi Science Centre, Ural Branch, Russian Academy of Sciences, 28 Kommunisticheskaya St., 167982 Syktyvkar, Russia; kateplus@mail.ru (E.P.); yushelen77@gmail.com (E.Y.); lyubov.schilova@yandex.ru (L.K.); kukushonok90@yandex.ru (N.Z.); dobrovolskaya.evgenia@gmail.com (E.S.); ilyasolovev-ksc@yandex.ru (I.S.); dashka-konst@yandex.ru (D.Y.); mnr270496@gmail.com (N.P.); ulyasheva-1309@yandex.ru (N.U.); mshaposhnikov@mail.ru (M.S.); 2 Institute of Natural Sciences, Pitirim Sorokin Syktyvkar State University, 55 Oktyabrsky Prosp., 167001 Syktyvkar, Russia; 3 Laboratory of Post-Genomic Research, Engelhardt Institute of Molecular Biology, Russian Academy of Sciences, 32 Vavilov St., 119991 Moscow, Russia

**Keywords:** lifespan, aging, radioresistance, ionizing radiation, *Agronaute*, *piwi*, *Drosophila melanogaster*

## Abstract

Small RNAs are essential to coordinate many cellular processes, including the regulation of gene expression patterns, the prevention of genomic instability, and the suppression of the mutagenic transposon activity. These processes determine the aging, longevity, and sensitivity of cells and an organism to stress factors (particularly, ionizing radiation). The biogenesis and activity of small RNAs are provided by proteins of the Argonaute family. These proteins participate in the processing of small RNA precursors and the formation of an RNA-induced silencing complex. However, the role of Argonaute proteins in regulating lifespan and radioresistance remains poorly explored. We studied the effect of knockdown of *Argonaute* genes (*AGO1*, *AGO2*, *AGO3*, *piwi*) in various tissues on the *Drosophila melanogaster* lifespan and survival after the γ-irradiation at a dose of 700 Gy. In most cases, these parameters are reduced or did not change significantly in flies with tissue-specific RNA interference. Surprisingly, *piwi* knockdown in both the fat body and the nervous system causes a lifespan increase. But changes in radioresistance depend on the tissue in which the gene was knocked out. In addition, analysis of changes in retrotransposon levels and expression of stress response genes allow us to determine associated molecular mechanisms.

## 1. Introduction

Lifespan is determined by the processes that occur at the molecular, cellular, tissue, and organism levels, as well as the influence of damaging environmental factors and other external conditions. Among the molecular mechanisms of lifespan regulation, epigenetic mechanisms have a special place. On the one hand, it provides the implementation of hereditary information embedded in the cells of an organism. On the other hand, it is necessary for fine-tuning the gene expression in accordance with the entering of exogenous stimuli. The well-coordinated work of these two processes maintains the vitality of an organism and ensures its longevity. However, a disturbance of epigenetic regulation can lead to cumulative negative consequences associated with the loss of functionality of cells and an organism, a decrease in its adaptive capabilities [1,2]. This is exactly what happens during the aging of an organism, therefore, epigenetic alterations are one of the basic hallmarks of aging [3]. During aging, there is a change in the structure of chromatin (for example, a loss of nucleosomes and a decrease in the amount of heterochromatin), DNA methylation status, modification of histone marks, changes in the patterns of noncoding RNA activity, epigenetic drift [4]. In addition to the fact that such changes lead to a disturbance of gene expression, they also cause a number of other fatal consequences. For example, the loss of heterochromatin, DNA hypomethylation, and changes in histone labels lead to the activation of the expression of silent transposable elements (or transposons), which increases the accumulation of DNA damages and mutations, and causes genome instability [2,4,5]. Epigenetic dysregulation contributes to the pathogenesis of age-related pathologies, such as cancer, atherosclerosis, type 2 diabetes, mental and neurodegenerative diseases, and a decrease in the immune response [6,7].

Among epigenetic mechanisms, small RNAs are required to coordinate many cellular processes, including post-transcriptional regulation of gene expression, regulation of heterochromatin formation, prevention of genome instability, and suppression of the mutagenic activity of transposons [8,9,10,11]. Small RNAs include three classes and differ in the mechanism of their biogenesis and the type of protein with which they are associated. These are endogenous short interfering RNAs (endo-siRNAs) that are targeted on mRNA and transposons, microRNAs (miRNAs) that regulate mRNA expression, and P-element induced wimpy testis (PIWI)-interacting RNAs (piRNAs) that are essential for the suppression of transposons’ activity. In addition, there are exogenous short interfering RNAs (exo-siRNAs) that are derived from viral double-stranded RNAs (dsRNAs) or artificial dsRNAs, and are aimed to restrict viral and external activity [8,9,10,12]. They are important for coordinating the organism development, forming various organs and tissues, controlling metabolism, and maintaining genome integrity [8,9,10]. Moreover, there is evidence for the important role of small RNAs in regulating lifespan and providing resistance to a range of environmental stressors [2,11,12,13,14,15,16].

The biogenesis and activity of small RNAs are provided by proteins of the Argonaute family. These proteins are involved in the processing of small RNA precursors and the formation of the RNA-induced silence complex (RISC). At the same time, an Argonaute protein loaded with a mature small RNA forms active RISC, which targets a corresponding molecule (mRNA or transposon), carries out its catalytic degradation, and inhibits translation [9,17]. Previous studies have demonstrated the role of some genes of the *Argonaute* family in the lifespan regulation. For example, it was found that in *Caenorhabditis elegans* the *alg-1* and *alg-2* genes conversely regulate lifespan: *alg-1* promotes longevity, while *alg-2* limits lifespan. This is mediated by their different roles in the regulation of DAF-2/insulin/IGF-1 and DAF-16/FOXO signaling pathways [18]. In *Drosophila melanogaster*, a mutation in the *AGO2* gene leads to a significant reduction in lifespan, which is associated with an increase in transposon expression in the brain and age-dependent memory impairment [19]. In addition, the activity of genes of small RNA biogenesis, including the *Argonaute* family, mediates beneficial effects of pro-longevity interventions, such as intermittent fasting [20]. However, there is almost no data on the effects of partial downregulation or tissue-specific knockdown of *Argonaute*.

It should be noted that the range of studied functions of small RNAs and proteins responsible for their biogenesis is currently expanding. In particular, it is known that some miRNAs (as well as lncRNAs) are involved in the response to DNA damage and DNA repair, due to participating in the network of signaling pathways [21,22,23]. At the same time, disruption of the activity of small RNA biogenesis genes and proteins encoded by them (such as AGO2 and PIWIL2) reduces the survival of human cells after exposure to genotoxic impacts (such as UV light, ionizing radiation, and others) as a result of distorted regulation of cell cycle, apoptosis, and DNA repair [24,25,26]. Similar data were obtained in an in vivo model of *Caenorhabditis elegans* with a mutation in the *alg-2* gene [27]. In addition, the role of complexes of double-strand break-induced small RNAs (diRNAs) and AGO2 (diRISCs) in the repair of double-stranded DNA breaks (mainly via homologous recombination) has been described [28,29]. Thus, small RNAs and Argonaute proteins are important for the response to DNA damage, and apparently, play a significant role in the response of cells and an organism to genotoxic agents.

Thus, it is obvious that the Argonaute proteins (as well as the small RNAs associated with them) are involved in regulating lifespan and the organism’s resistance to radiation. However, these functions remain poorly understood and require investigation. The fruit fly *Drosophila melanogaster* is an appropriate model for this task. Its genome contains five genes encoding proteins of the Argonaute family. Among them, the Argonaute subfamily includes AGO1 (provides maturation and functioning of miRNAs) and AGO2 (performs biogenesis and specifically binds to siRNAs). The PIWI subfamily includes piwi, AGO3, and Aubergine, which are required for piRNA processing and functioning [30,31]. Thus, Argonaute proteins in *Drosophila* are relatively specific for types of small RNAs, which makes it possible to analyze the contribution of biogenesis and the functioning of each to the studied processes. In addition, fruit fly as a model animal has known advantages, due to their short life cycle, maintenance availability, accessibility of genetic interventions, and evolutionary conservatism of many signaling pathways and genes [32].

In the present work, we studied for the first time the effect of *Argonaute* genes’ knockdown (*AGO1, AGO2, AGO3, piwi*) in various tissues on *Drosophila melanogaster* lifespan and survival after the γ-irradiation at a dose of 700 Gy. In addition, changes in the levels of retrotransposons and expression of stress response genes were analyzed to determine the molecular mechanisms involved. It was previously found that genes of antioxidant defense, DNA damage response, and repair play a critical role in both lifespan regulation and the reaction of cells, tissues, and a whole organism to ionizing irradiation [33,34,35,36,37,38,39,40,41]. In addition, genes involved in different mechanisms of proteostasis demonstrate changes during aging and after irradiation as well [38,39,42,43]. Therefore, we analyses the expression levels of genes from each group.

## 2. Results

### 2.1. Effects of Down-Regulation of Argonaute Genes on the Drosophila Lifespan

**Figure 1:**
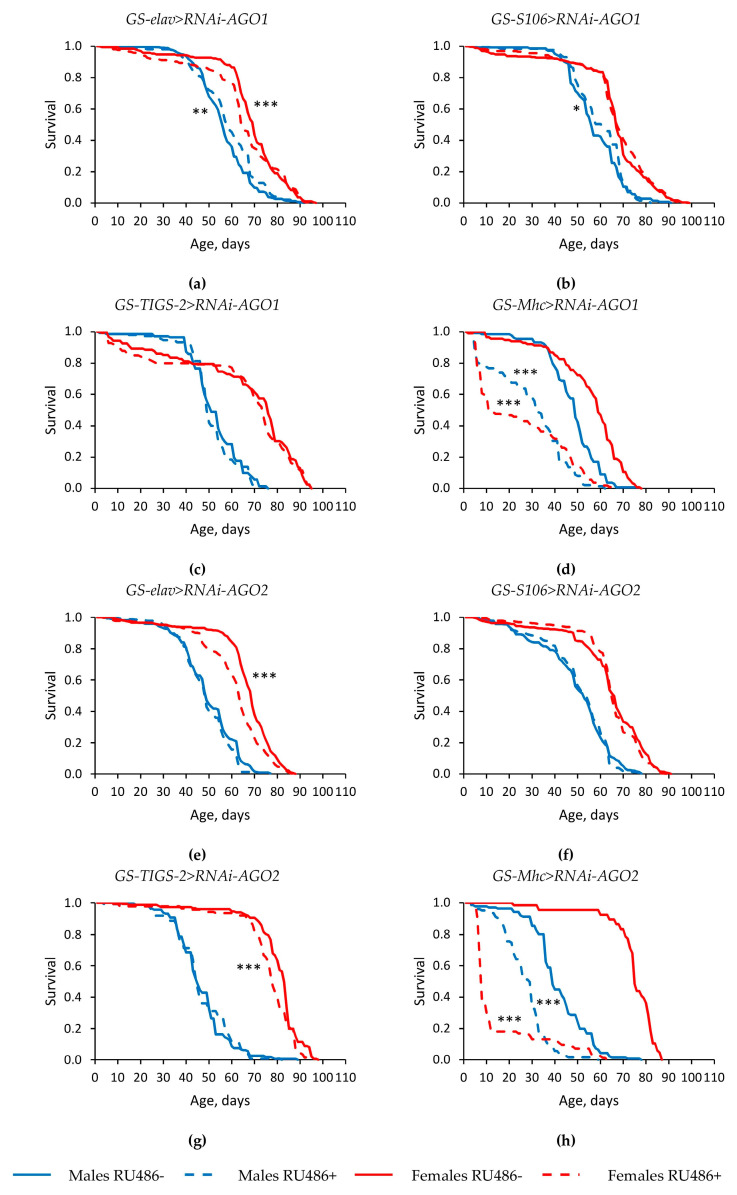
Influence of *AGO1* (**a**–**d**) and *AGO2* (**e**–**h**) knockdown in the nervous system (**a**,**e**) (two replicates combined), fat body (**b**,**f**) (two replicates combined), guts (**c**,**g**), muscles (**d**,**h**) on the survival of *Drosophila melanogaster*. Differences between survival curves of flies with *Argonaute* genes’ knockdown (RU486+) and without knockdown (RU486-) are statistically significant with * *p* < 0.05, ** *p* < 0.01, *** *p* < 0.001 (Kolmogorov–Smirnov test).

**Figure 2:**
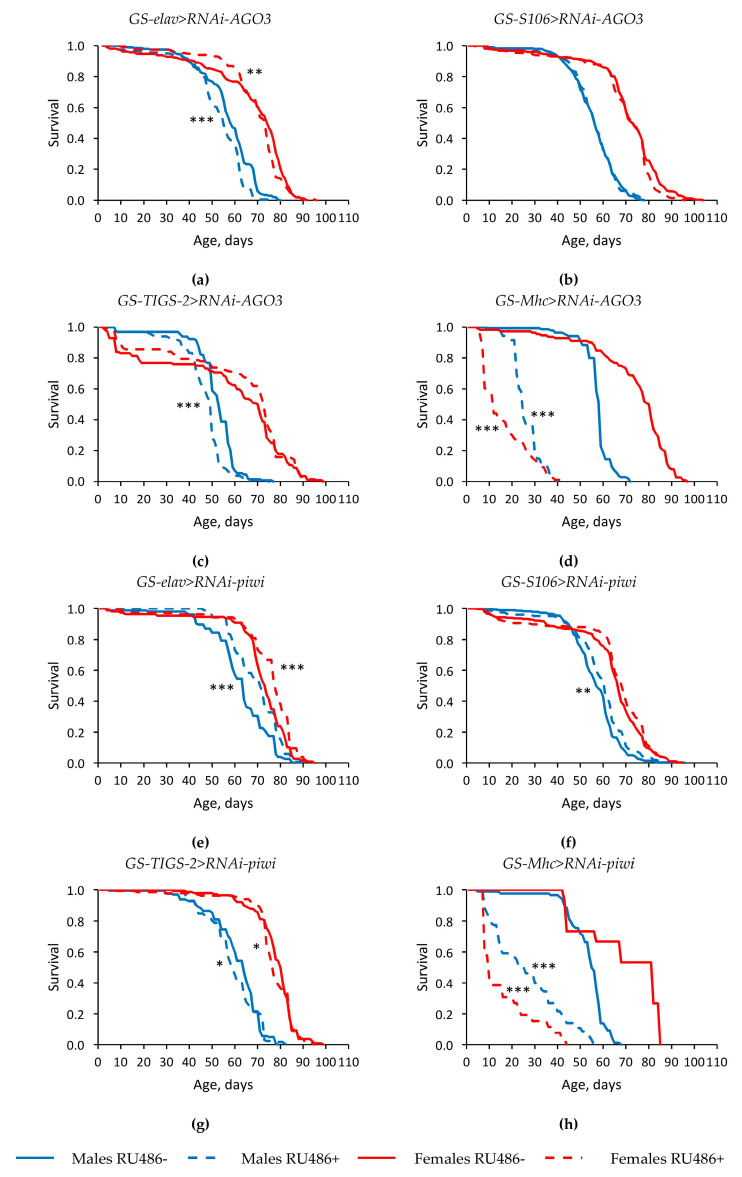
Influence of *AGO3* (**a**–**d**) and *piwi* (**e**–**h**) knockdown in the nervous system (**a**,**e**) (two replicates combined), fat body (**b**,**f**) (two replicates combined), guts (**c**,**g**), muscles (**d**,**h**) on the survival of *Drosophila melanogaster*. Differences between survival curves of flies with *PIWI* genes’ knockdown (RU486+) and without knockdown (RU486-) are statistically significant with * *p* < 0.05, ** *p* < 0.01, *** *p* < 0.001 (Kolmogorov–Smirnov test).

Tissue-specific knockdown of genes of the *Argonaute* family in most cases either did not have a statistically significant effect, or led to a decrease in the median lifespan (by 3.0–89.3%, *p* < 0.05) and the parameter of maximum lifespan (the age of 90% mortality) (by 3.1–62.9%, *p* < 0.05) in *Drosophila* males and females (Figure 1 and Figure 2, Table S1). However, in some replicates of the experiment, the studied longevity parameters were increased in flies with RNA interference of the *AGO1* and *AGO3* genes. Moreover, the median lifespan was reproducibly higher in males and females with *piwi* knockdown in the nervous system and in the fat body compared with flies without induction of RNA interference (by 2.1–12.5%, *p* < 0.05) (Figure 2e,f, Table S1).

Since the positive effect of knockdown of some of the studied *Argonaute* genes on longevity was manifested in the case of their RNA interference in the nervous system and the fat body, we carried out further research only with these variants.

### 2.2. Radioresistance of Drosophila with Knockdown of Argonaute Genes

**Figure 3:**
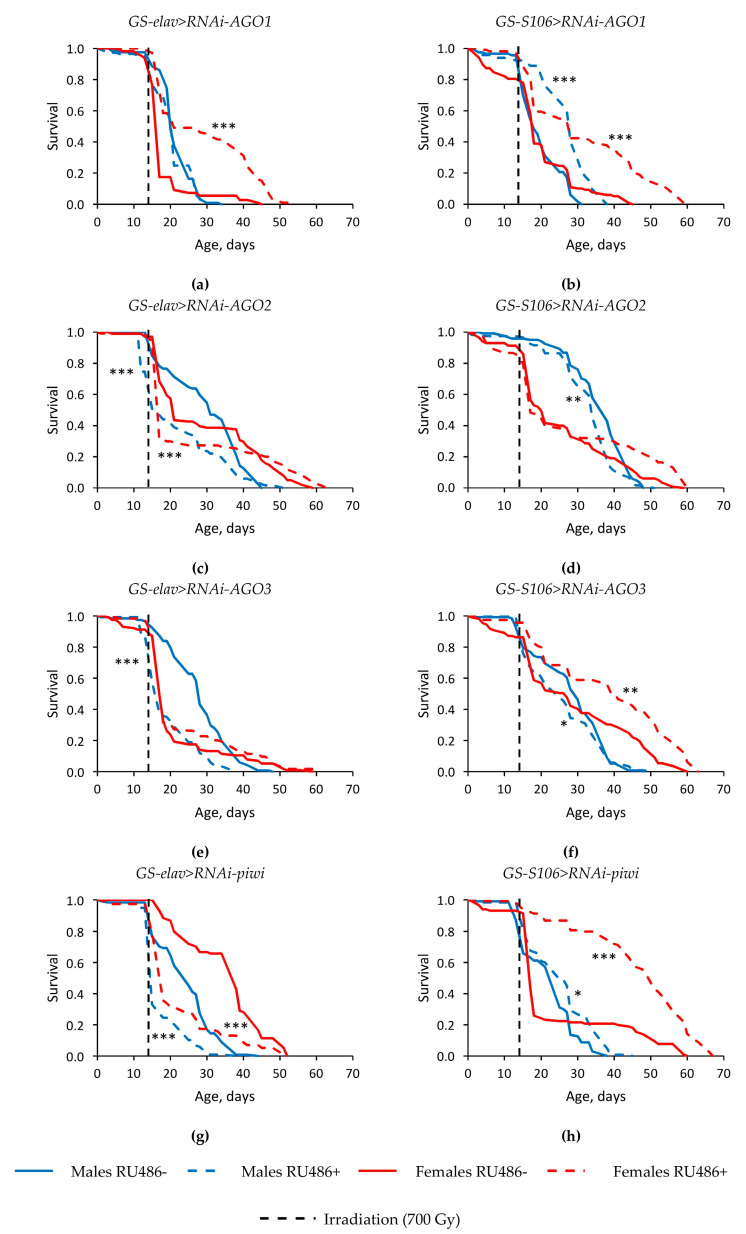
Influence of *AGO1* (**a**,**b**), *AGO2* (**c**,**d**), *AGO3* (**e**, **f**), *piwi* (**g**,**h**) knockdown in the nervous system (**a**,**c**,**e**,**g**) and fat body (**b**,**d**,**f**,**h**) on the survival of *Drosophila* flies after γ-irradiation at the dose of 700 Gy. Differences between survival of flies curves with *Argonaute* knockdown (RU486+) and without knockdown (RU486-) are statistically significant with * *p* < 0.05, ** *p* < 0.01, *** *p* < 0.001 (Kolmogorov–Smirnov test).

The exposure to γ-irradiation at a dose of 700 Gy extremely reduced the survival of *Drosophila* in both sexes, regardless of the tissue-specific expression of the *Argonaute* family genes. The median survival was decreased by 17.4–76.7% (*p* < 0.001), and the age of 90% mortality was lower by 18.8–73.8% (*p* < 0.001) in irradiated flies compared to non-irradiated ones (Figure 3, Table S2).

In the most experimental variants, tissue-specific knockdown of genes of the *Argonaute* family negatively affected the radioresistance of *Drosophila* of both sexes, decreased the median survival (by 10.5–55.3%, *p* < 0.001) and the maximum survival rate (by 21.1–23.5%, *p* < 0.001) in conditions of γ-irradiation (Figure 3, Table S2). However, flies of both sexes with RNA interference of the *AGO1* and *piwi* genes in the fat body (Figure 3b,h), females (but not males) with RNA interference of *AGO2* and *AGO3* in the fat body (Figure 3b,h), and females with *AGO1* neuronal knockdown showed a high resistance to the radiation exposure (Figure 3a). In these variants of the experiment, the median survival rate was increased by 23.5–200% (*p* < 0.001), the age of 90% mortality was higher by 17.6–123.8% (*p* < 0.001) compared with irradiated flies without induction of RNA interference.

It should be noted that an increase in lifespan did not coincide in all cases with an increase in radioresistance. In particular, flies with reduced *piwi* activity in the nervous system under irradiation conditions had a reduced survival rate compared to variants without induction of RNA interference (Figure 2e and Figure 3g).

### 2.3. Age-Related Changes in the Expression of Argonaute Genes, Retrotransposons, and Stress Response Genes

**Figure 4:**
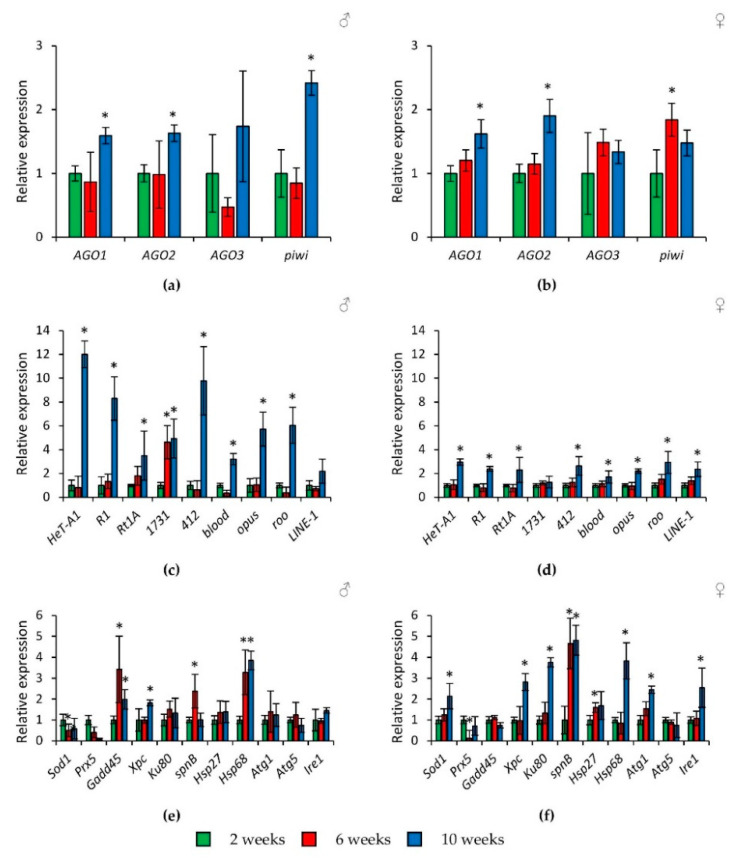
Age-related changes in the expression of *Argonaute* genes (**a**,**b**), transposable elements (**c**,**d**), and stress response genes (**e**,**f**) in wild-type *Canton-S* males (**a**,**c**,**e**) and females (**b**,**d**,**f**). Differences between relative expression levels of the investigated genes at the age of 2 weeks and at the ages of 6 and 10 weeks are statistically significant with * *p* < 0.05 (Mann-Whitney U-test).

In flies of the wild-type *Canton-S* strain, a slight increase in the expression of genes of the *Argonaute* family (by 1.6–2.4 times, *p* < 0.05) and a pronounced activation of retrotransposons (by 1.7–12.0 times, *p* < 0.05) at the age of 10 weeks was observed (Figure 4a–d, Tables S3 and S4). It should be noted that this tendency was repeated separately in the abdomens of *Drosophila*, and in part, in the heads (but not in the thoraxes) (Figures S1 and S2, Tables S3 and S4).

In addition, at the ages of 6 and 10 weeks, flies had increased transcription of some stress response genes both in whole bodies and in individual parts of the body (Figure 4e,f and Figure S3, Tables S3 and S4). In particular, activation (by 1.8–15.2 times, *p* < 0.05) is shown for genes of response and repair of DNA damages (*Gadd45, Xpc, Ku80, spn-B*) and proteostasis genes (*Hsp27, Hsp68, Atg1, Ire1*). At the same time, the activity of the *Prx5* gene was decreased.

### 2.4. Changes in Expression Levels of Retrotransposons Associated with Argonaute Genes’ Knockdown and γ-Irradiation

**Figure 5:**
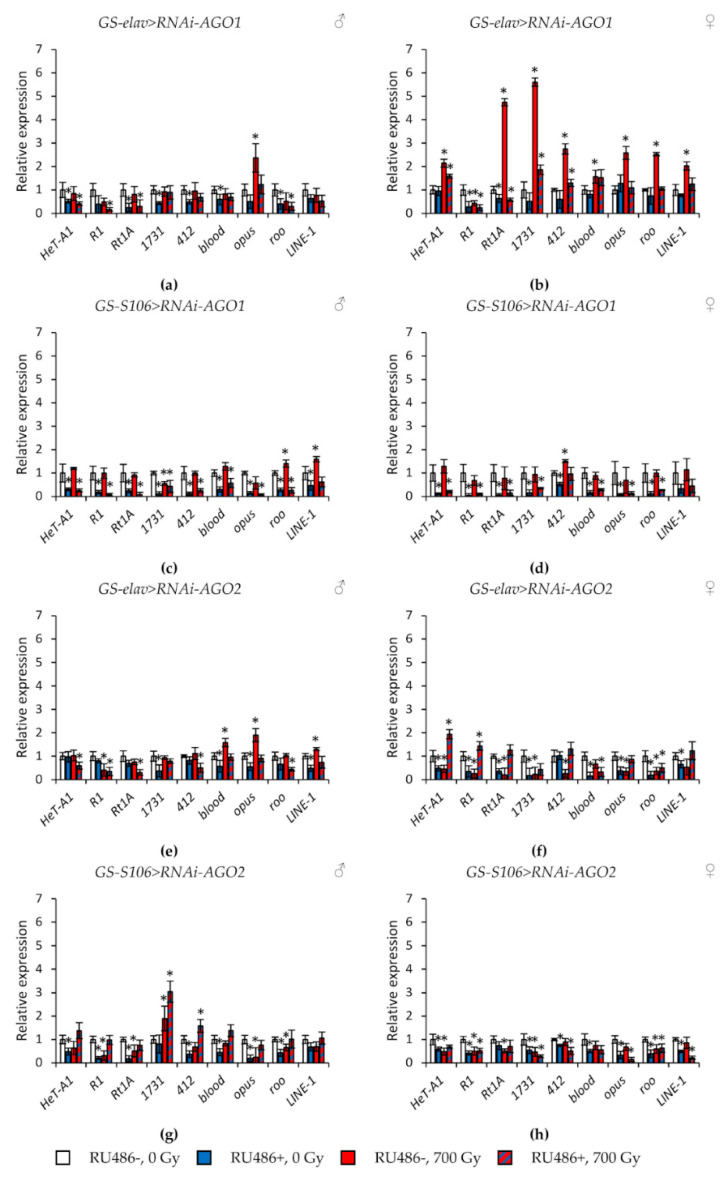
Changes in the expression of retrotransposons in irradiated and unirradiated males (**a**,**c**,**e**,**g**) and females (**b**,**d**,**f**,**h**) with *AGO1* (**a**–**d**) and *AGO2* (**e**–**h**) knockdown. Differences between relative expression levels of retrotransposons of unirradiated flies without *Argonaute* genes’ knockdown (RU486-, 0Gy) and each of other experimental variants are statistically significant with * *p* < 0.05 (Mann-Whitney U-test).

**Figure 6:**
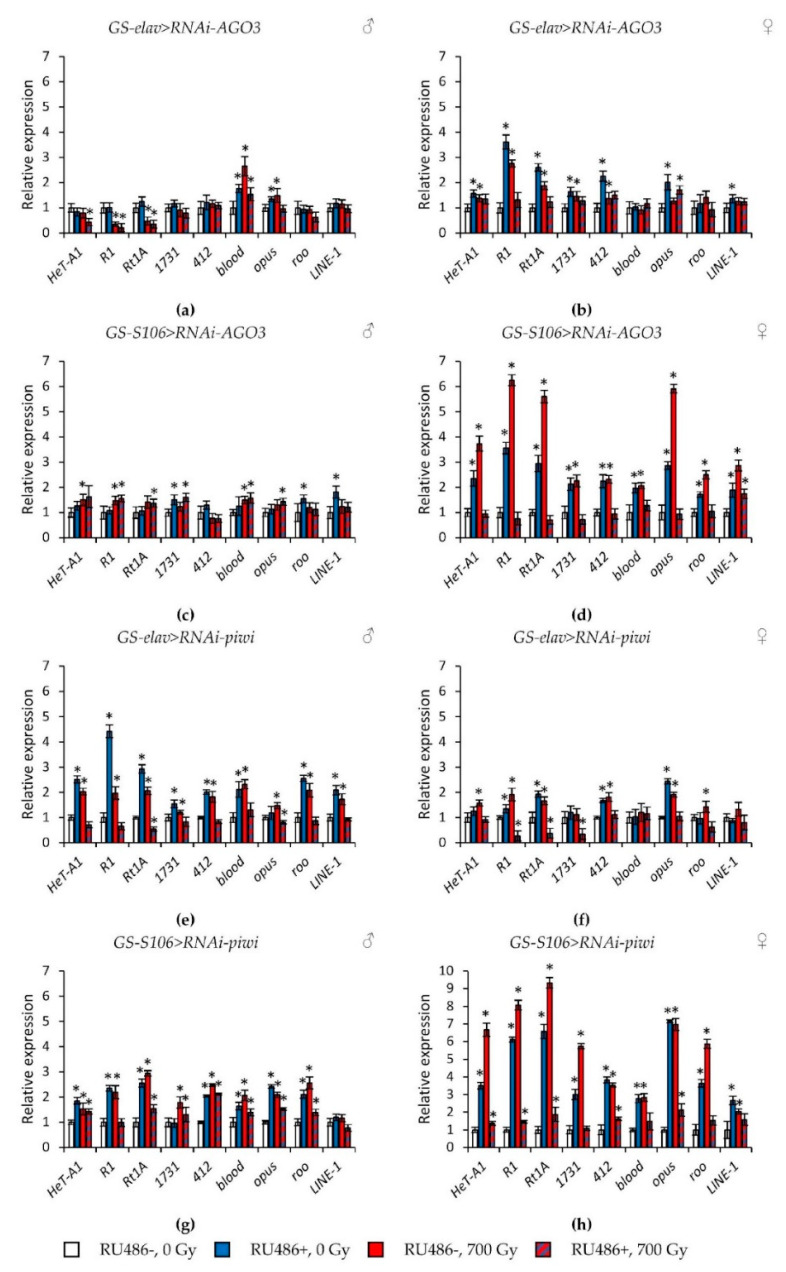
Changes in the expression of retrotransposons in irradiated and unirradiated males (**a**,**c**,**e**,**g**) and females (**b**,**d**,**f**,**h**) with *AGO3* (**a**–**d**) and *piwi* (**e**–**h**) knockdown. Differences between relative expression levels of retrotransposons of unirradiated flies without *PIWI* genes’ knockdown (RU486-, 0Gy) and each of other experimental variants are statistically significant with * *p* < 0.05 (Mann-Whitney U-test).

In *Drosophila* without the induction of RNA interference of genes of the *Argonaute* family, γ-irradiation at a dose of 700 Gy caused the activation of retrotransposons (by 1.3–9.3 times, *p* < 0.05), or did not lead to statistically significant changes (Figure 5 and Figure 6, Tables S5–S8). Exceptions are flies with the genotypes *GS-elav > RNAi-AGO2* and *GS-elav>RNAi-AGO2*, in which radiation exposure suppressed the retrotransposons’ expression in some cases (Figure 5e–h, Table S6).

At the same time, knockdown of genes of the subfamilies *Argonaute* and *PIWI* had different effects on the activity of retrotransposons. In flies with knockdown of genes of the *Argonaute* subfamily (*AGO1* and *AGO2*) in the nervous system and the fat body both under γ-irradiation and without irradiation, the activity of retrotransposons decreased by 1.4–22.1 times (*p* < 0.05) compared with the variants without induction of RNA interference (Figure 5, Tables S5 and S6). RNA interference of genes of the *Piwi* subfamily (*AGO3* and *piwi*) significantly increased the activity of retrotransposons (by 1.3–9.3 times, *p* < 0.05) in unirradiated flies. However, γ-irradiation, on the contrary, reduced the activity of transposable elements in males and females with knockdown of *PIWI* genes by 1.2–8.2 times (*p* < 0.05) compared with irradiated flies without induction of RNA interference (Figure 6, Tables S7 and S8).

### 2.5. Changes in Expression Levels of Stress Response Genes Associated with Argonaute Genes’ Knockdown and γ-Irradiation

**Figure 7:**
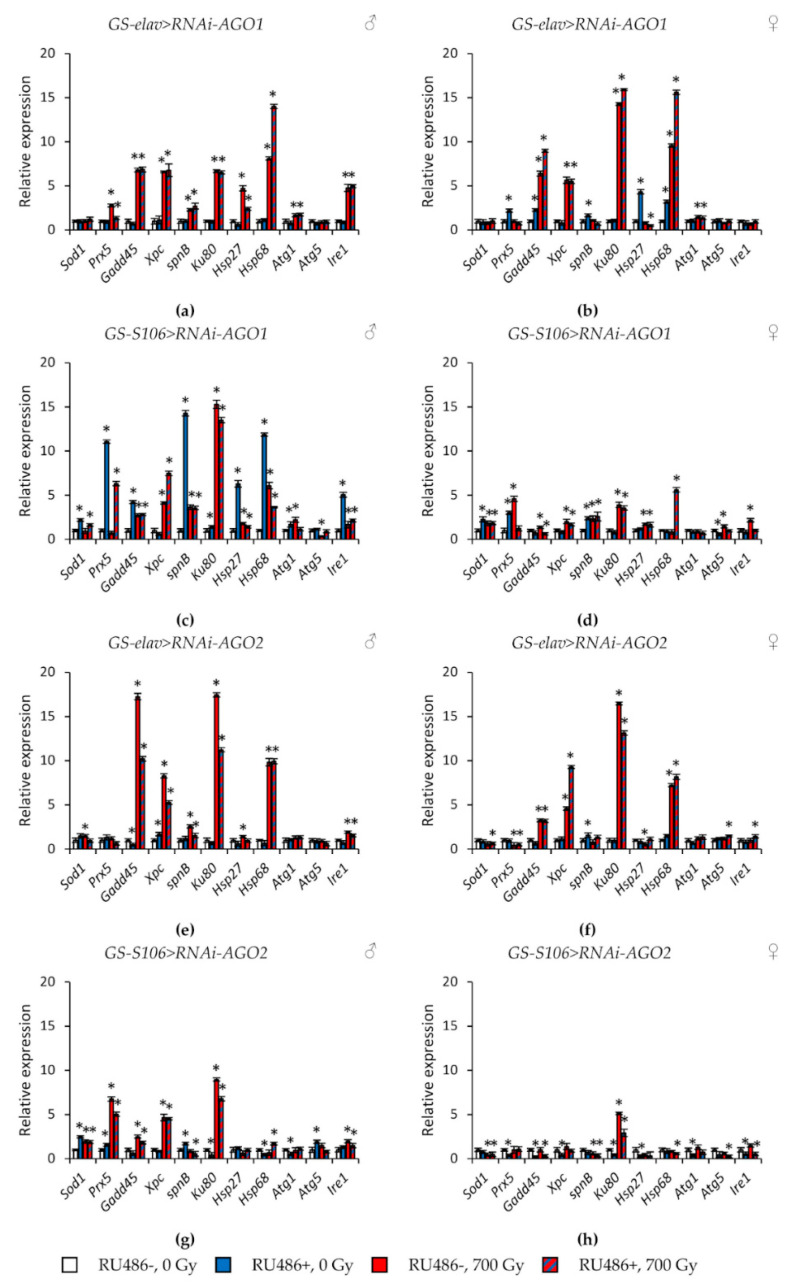
Changes in the expression of stress response genes in irradiated and unirradiated males (**a**,**c**,**e**,**g**) and females (**b**,**d**,**f**,**h**) with *AGO1* (**a–d**) and *AGO2* (**e–h**) knockdown. Differences between relative expression levels of the investigated genes of unirradiated flies without *Argonaute* genes’ knockdown (RU486-, 0Gy) and each of other experimental variants are statistically significant with * *p* < 0.05 (Mann-Whitney U-test).

**Figure 8:**
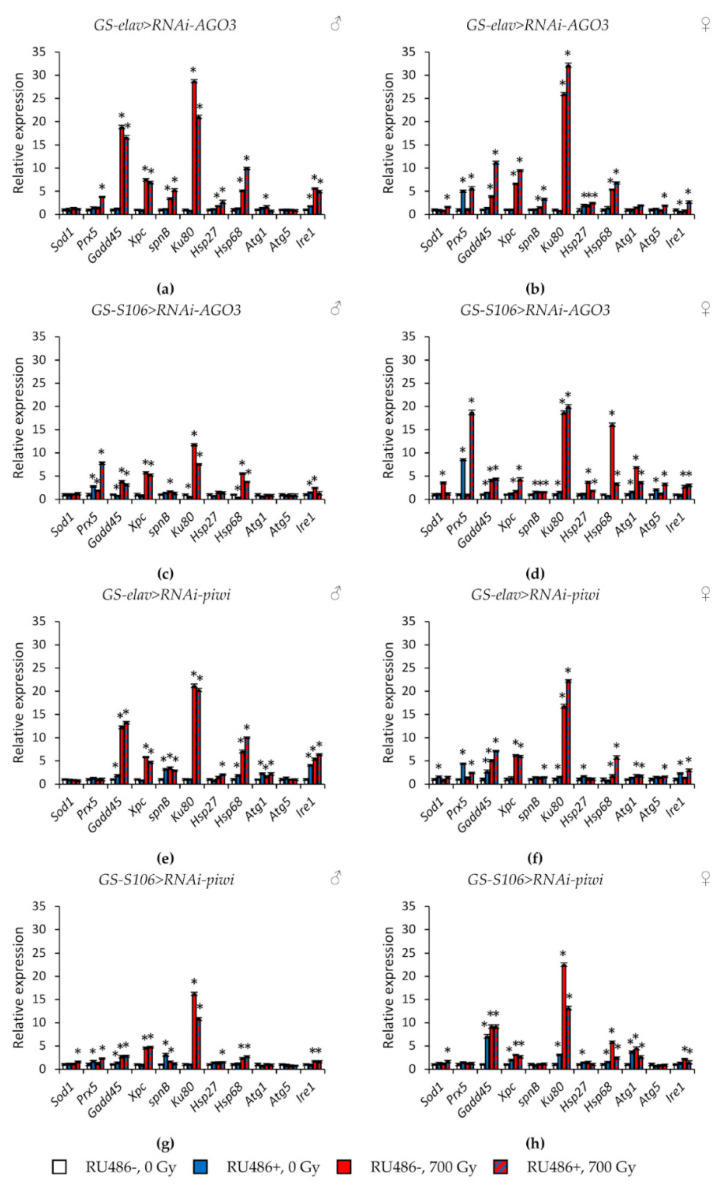
Changes in the expression of stress response genes in irradiated and unirradiated males (**a**,**c**,**e**,**g**) and females (**b**,**d**,**f**,**h**) with *AGO3* (**a**–**d**) and *piwi* (**e**–**h**) knockdown. Differences between relative expression levels of the investigated genes of unirradiated flies without *PIWI* genes’ knockdown (RU486-, 0Gy) and each of other experimental variants are statistically significant with * *p* < 0.05 (Mann-Whitney U-test).

Tissue-specific knockdown of the *AGO1* gene caused the greatest activation of stress response genes. In particular, the expression of genes of antioxidant defense (*Sod1, Prx5*), genes of DNA damage response and repair (*Gadd45, spn-B*), genes of heat shock proteins (*Hsp27, Hsp68*) was increased by 1.7–14.3 times (*p* < 0.05) (Figure 7a–d, Table S5). The most pronounced induction of their activity was observed in males with *AGO1* RNA interference in the fat body. In addition to these genes, *Ku80, Atg1,* and *Ire1* were also activated in this variant of the experiment (Figure 7c). Similar but less pronounced changes were observed in flies with *piwi* knockdown in the nervous system and the fat body (Figure 8e–h, Table S8). At the same time, the decreased activity of *AGO2* and *AGO3* mainly decreased the activity of stress response genes (Figure 7e–h and Figure 8a–d, Tables S6 and S7).

γ-Irradiation led to a significant activation (by 1.4–32.2, *p* < 0.05) of genes responsible for the response to genotoxic stress (*Gadd45, Xpc, Ku80*) and proteotoxic stress (*Hsp68*). This effect was observed both in variants with RNA interference of *Argonaute* genes and without induction of RNA interference (Figure 7 and Figure 8, Tables S5–S8). Other studied stress response genes were also activated in some variants of the experiment, but to a lesser extent.

## 3. Discussion

Aging is accompanied by age-related differential changes in the expression of small RNAs, which is closely associated with impaired biogenesis and regulation. Dysregulation of small RNA biogenesis proteins and corresponding changes in the functioning of miRNAs, siRNAs, and piRNAs lead to a global disruption of gene expression and chromatin structure with subsequent negative consequences at the molecular, cellular, tissue, and organismal levels. For example, they include loss of genome integrity and genetic instability, impaired stress response, metabolism, immunity, regenerative abilities, increased inflammatory responses, and others. Such changes significantly deplete the organism’s life support systems, cause age-related disorders and aging [4,44].

During aging, depending on the tissue and physiological state of the organism, both a critical decrease in the expression of small RNA biogenesis proteins and their excessive activation can occur. Predominantly, aging human cell cultures, as well as cells obtained from old donors, are characterized by reduced activity of small RNA biogenesis genes, such as *Drosha, Dicer, Exportin 5,* and *AGO2*. Such changes are accompanied by shifts in the expression patterns of miRNAs [45,46,47,48]. Similar data were obtained in studies of age-related changes in various tissues of rodents [47,48,49] and in nematodes [48]. Nevertheless, some data indicate the nonlinear pattern in the dynamics of the activity of genes encoding enzymes of small RNA biogenesis. Thus, in the hearts of rats, *AGO1* and *AGO2* firstly increase the expression, but at the end of life, they decrease it [50]. In addition, it should be noted that the levels of small RNAs not only depend on the activity of proteins of its biogenesis, but the feedback loop is observed. For example, a miRNA-directed mechanism of age-related changes in the expression of an *Argonaute* gene has been described using the *Caenorhabditis elegans* model. In particular, *miR-71*, which is activated during aging, suppresses *alg-1* and limits the lifespan of nematodes [14].

In this work, the analysis of gene expression showed that there is an age-related increase in the expression of the *Argonaute* family genes in whole *Drosophila* bodies (total homogenate), in heads and abdomens (but not in thoraxes). At the same time, increased activity was also observed in retrotransposons and particular stress response genes. In other words, despite the fact that aging flies activate mechanisms aimed at the piRNAs, miRNAs, and siRNAs production, which suppress the activity of transposable elements and target mRNAs, we did not observe the corresponding effect.

We assume at least two explanations for the data obtained. First, an increase in the transcriptional activity of the *Argonaute* genes does not indispensably indicate an increase in the level of the corresponding proteins and their functional activity. Deregulation of their activity may occur at post-transcriptional levels. For example, it was found that a decrease in *AGO2* mRNA methylation in human cells during aging takes place, probably leading to deregulation of miRNA expression [45]. Second, an increase in the activity of the *Argonaute* genes may be a manifestation of a compensatory response to the increasing age-related activity of retrotransposons, disruption of the heterochromatin structure, and cellular stress. This may also be the reason for the activation of stress response genes in old fruit flies. Indeed, the chronic activation of stress-sensitive pathways during aging has been previously described. In several experimental models, the induction of stress response genes was found both in individual organs and throughout the body [51,52,53,54]. In the early stages of aging or in the case of a short period of time after an acute damaging impact, this tendency can provide faster recovery and better survival of an organism. However, the chronic activation and dysregulation of the stress-sensitive pathways during aging causes homeostasis destruction and energy depletion. There is a general decrease in the efficiency of cellular and organismal responses to stressful influences, a decrease in the work of repair systems, an increase in the number of senescent and malfunctioning cells, and other destructive processes [53,55,56,57].

An increase in the activity of retrotransposons (which we observed in the experiment) is both a consequence of age-related deregulation of the mechanisms maintaining the heterochromatin in the condensed state and preserving cellular defense, and the cause of genotoxic stress with the subsequent development of degenerative processes [5,58]. Earlier it has been found that the activity of transposable elements increases in various organs of aging animals. For example, such changes have been shown in the brain [19] and the adipose body of fruit flies [59]. The changes observed are accompanied by an age-related loss of the organs’ functions.

In the present research, we studied the effect of knockdown of the *Argonaute* genes in various tissues on the *Drosophila melanogaster* lifespan. Based on the data described above, we assumed two possible consequences of the *Argonaute* knockdown. Firstly, a decrease in the activity of the *Argonaute* genes forces up age-related changes in the tissues of flies as a result of small RNA deregulation, and leads to a lifespan shortening. Secondly, suppression of the *Argonaute* genes partly restores the imbalance in the abundance and functioning of the proteins translated from them and diminishes age-related hyperactivation, at the level of retaining energy resources at least.

We found that decreased activity of genes in the *Argonaute* family causes changes in the lifespan depending on a gene and the tissue in which a gene was knocked down. In most cases, tissue-specific RNA interference of genes of the *Argonaute* family either did not have a statistically significant effect, or led to a shortened lifespan, which is consistent with the first hypothesis. It is worth noting that there are few studies where the reduced activity of the *Argonaute* genes also led to a lifespan reduction in model animals. For example, in *Drosophila melanogaster*, mutations in the *AGO2* gene resulted in a progressive deterioration in the functions of the nervous system and a lifespan decrease [19]. At the same time, *piwi* mutations lead to opposite effects on the lifespan and health of fruit flies, depending on the allele. *Drosophila* with a heterozygous *piwi^2^* mutation had a short lifespan, increased sensitivity to starvation, and reduced immunity. In the fat body of flies, the *piwi^2^* mutation caused a decrease in the level of piRNAs, activation of transposable elements, an increase in DNA damages, and a loss of lipid stores [60]. However, the *piwi^c362^* mutation led to an increase in lifespan [61]. In our study, RNA interference of the *piwi* gene in the nervous system and the fat body, as well as knockdown of the *AGO1* and *AGO3* genes in individual cases, also led to an increase in the lifespan. These data are consistent with the second hypothesis and indicate a critical role in the emerging epigenetic imbalance in the biogenesis mechanisms of miRNAs and piRNAs, but not siRNAs. Activated Argonaute proteins can enhance gene repression (as a response to an increase in the proportion of heterochromatin and activation of transposons), and at the same time, suppress the activity of genes important for survival. Indeed, it has been found that proteins of the Argonaute family in nematodes [18] and piwi in fruit flies [62] affect the DAF-16/FOXO and DAF-2/IGF-1/insulin signaling pathways, which regulate longevity and aging. In our study, knockdown of *AGO1* and *piwi* genes in the nervous system and the fat body caused activation of stress response genes, especially antioxidant defense genes (*Sod1, Prx5*), genes of DNA damage response and repair (*Gadd45, spn-B*), and genes encoding heat shock proteins (*Hsp27, Hsp68*). Several of these genes have been identified previously as pro-longevity genes [33,34,35,42,43]. At the same time, suppression of *AGO2* and *AGO3* expression mainly reduced the activity of stress response genes.

At the same time, unexpected data were obtained on the effect of RNA interference of *Argonaute* genes on the activity of retrotransposons. Since the genes of the *PIWI* subfamily are an important part of the mechanism for controlling the activity of transposons, a disruption of their regulation leads to a surge in the activity of these genetic elements. This effect we observed in flies with tissue-specific knockdown of *AGO3* and *piwi*. Surprisingly, knockdown of genes of the *Argonaute* subfamily (*AGO1* and *AGO2*), on the contrary, reduced the activity of retrotransposons. The mechanism of siRNAs is also aimed at suppressing the activity of transposable elements; mutations in *AGO2* were previously found to increase their expression in the *Drosophila* brain [19]. Despite the fact that it is not clear how the knockdown of *AGO1* and *AGO2* reduces the activity of retrotransposons in our experiment, it is obvious that the change in the activity of these genetic elements did not play a key role in the observed effects.

Currently, there are a little data regarding the contribution of the activity of *Argonaute* genes to age-related changes and lifespan regulation, therefore we can only assume the mechanisms of the lifespan effects of tissue-specific *Argonautes*’ knockdown. It is known that AGO2 takes over part of the functions of AGO1 in aging fruit flies. Deep sequencing of small RNAs revealed a global increase in miRNAs loaded into AGO2, but not AGO1, with age. This process is mediated by an increase in the level of 2′-O-methylation of miRNAs. Despite the fact that this mechanism is assumed to be associated with age-related events, its violation has even greater negative consequences. Thus, the *AGO2* mutation or the disruption of miRNA 2′-O-methylation leads to accelerated neurodegeneration and a reduction in the lifespan of flies [63]. Thus, the age-related activation of *AGO2* can be justified in connection with the increasing load on it; therefore, its knockdown caused rather a negative effect on the lifespan. At the same time, *AGO1* hyperactivation can enhance the growing imbalance with aging, so we observed the positive effects of its knockdown in some cases.

The piRNAs-PIWI mechanism was initially identified in germline cells. However, the understanding of the functions of piRNAs and proteins of the PIWI subfamily in somatic tissues and their role in regulating lifespan is now expanding [5,64]. For example, the regulation of transposon activity and the functioning of PIWI proteins is important for the maintenance of somatic stem cells and the prevention of aging-related tissue degeneration. Thus, it was shown that piwi is crucial for the suppression of age-related expression of transposons in stem cells of the *Drosophila* intestine and maintenance of epithelial homeostasis [65]. The piwi activity in the fat body is essential for regulating metabolism and the normal lifespan of flies [60]. A number of studies in rodents have established the role of PIWI proteins and piRNAs in the regeneration of axons of sensory neurons [66] and in the implementation of neuronal functions, for example, memory [67]. It is known that they not only regulate the formation of heterochromatin and break down transposable elements, but can also affect the activity of genes encoding proteins [66,68]. In addition, the activity of genes encoding PIWI proteins affects the fertility of model animals (their defects lead to infertility), determining age-related changes in reproductive abilities and affecting longevity [62,69].

We observed that neuronal knockdown of the *piwi* gene in males and females, as well as *AGO3* in females, increases the lifespan. Recent studies on *Drosophila melanogaster* have shown that the brain is characterized by genomic heterogeneity, and the mobility of retrotransposons is important for the activity of some parts of the brain. For example, transpositions in αβ neurons of mushroom bodies are important for the implementation of some functions, e.g., for memory. In these neurons, the activity of piRNA biogenesis proteins is reduced [70]. Thus, the elimination of age-related hyperactivation of the *piwi* and *AGO3* genes specifically in the nerve cells could lead to the preservation of the fly’s brain activity and an increase in the lifespan. In addition, *piwi* knockdown in the fat body of males also increased lifespan. But further research is required to identify possible mechanisms for this effect. Generally, divergency in the lifespan effects of *Argonaute* genes’ knockdown in different tissues may be associated with significant variations between gene expression profiles and the implementation of inherited information between tissues and cell types, as well as differences in proteome and metabolome composition [71,72,73,74].

Currently, data on the role of small RNA biogenesis proteins in the response of cells and an organism to the action of stress factors, as well as their interaction with proteins and signaling pathways of the stress response, are expanding. Previously, small RNAs called double-strand break-induced RNAs (diRNAs) have been identified. In human cells, they are loaded onto AGO2 (forming diRISC) and are important for triggering the repair of DNA double-strand breaks (mainly homologous recombination) by recruiting repair factors (particularly, Rad51) to target sites [28,29]. There are studies indicating the relationship of the AGO2 protein with isoforms of the transcription factor p53, one of the central regulators of the genotoxic stress response and the anticancer mechanisms. Studies on human cancer cell cultures have shown that p53 interacts (including indirectly through miRNAs) with AGO2 after DNA damage, affecting the biogenesis and activity of specific miRNAs. In turn, they can regulate the activity of p53 targets (such as GADD45A) and determine cellular processes, in particular, cell cycle arrest and apoptosis [75,76]. In addition, there is evidence of Argonaute- and miRNA-dependent mechanisms of regulation of the activity of other DNA damage response proteins, for example, ATM [23] and CDK [24]. It should be noted that proteins of the PIWI subfamily may also be involved in the repair of DNA damage caused by genotoxic agents, in particular, through the regulation of histone acetylation and chromatin relaxation [26].

For the Argonaute proteins’ functioning, they must interact with certain heat shock proteins. In particular, Hsp90 activity is required for the efficient targeting of AGO2 to processing bodies and stress granules, and also affects the production and functional activity of miRNAs and siRNAs [77]. Similarly, the association of the PIWI proteins with chaperones, for example, with the heat shock protein DNAJA1 in planarians, has been shown. Homologs of these proteins also interact in human gastric cancer cells [78]. In addition, the *Drosophila* organizing protein homolog Hsp70/90 (Hop) interacts with piwi and mediates the maintenance of genome stability in germline cells [79].

As indicated above, in our study, long-lived flies with knockdown of the *AGO1* and *piwi* genes in the nervous system and the fat body were characterized by activation of stress response genes. This effect also indicates the contribution of small RNA biogenesis genes to the stress response.

As a rule, changes in stress resistance, and in particular, radioresistance, corresponds to changes in lifespan. Thus, organisms that are more resistant to the action of negative environmental factors have higher viability and longevity [80]. We compared the survival rate of fruit flies under normal conditions and after acute exposure to γ-radiation. Nevertheless, the obtained data did not always correspond to the described pattern. For example, fruit flies with *AGO1* knockdown in the fat body and the nervous system and *piwi* knockdown in the fat body showed both increased lifespan and radioresistance. However, *piwi* RNA interference in the nervous system, which had a pro-longevity effect, significantly reduced the survival under irradiation conditions. RNA interference of *AGO2* and *AGO3* in the fat body of females did not significantly affect the lifespan of females under normal conditions, but increased the survival rate after γ-irradiation.

It should be noted that the relationship between radioresistance and the tissue in which an *Argonaute* gene was knocked out is more likely than the relationship with a particular gene. In general, flies with neuronal knockdown of the *Argonaute* genes were sensitive to the action of γ-radiation. It has been established that the brain in *Drosophila* is a highly sensitive organ to radiation exposure even at lower doses [81,82]. Thus, changes in the expression profile in neural tissues can critically affect radiosensitivity and overall survival. Our studies demonstrate that genes of the *Argonaute* family are necessary for the stability of the nervous system functioning under stressful conditions. At the same time, females (and to a lesser extent males) with knockdown of the *Argonaute* genes in the fat body, on the contrary, showed higher resistance to radiation. Previous data do not explain the observed effects. In contrast, *piwi* mutants exhibit piRNA depletion in the fat body, enhanced transposon mobilization, increased levels of DNA damage, decreased lipid stores, and increased stress sensitivity [60]. Mutations of *AGO2* and *PIWIL2* in human and rodent cells reduced their survival underexposure to UV light and ionizing radiation, and led to impaired responses to DNA damage [24,25,26]. Similar results were obtained for germ cells in irradiated *Caenorhabditis elegans* with loss of the *alg-2* gene. In this case, increased cell apoptosis associated with MAPK hyperactivation was observed [27]. In addition, studies on non-small cell lung cancer cells indicate no effect of *AGO2* gene knockdown on their radiosensitivity [83]. As in the case of differences in lifespan, opposite tissue-specific effects can be, due to significant differences in the profiles of transcriptomes, proteomes, and metabolomes in the tissues. However, pathways that determine the organismal radioresistance have their own specificity.

The data obtained for the activity of transposons and the expression of stress response genes also do not draw conclusions about the mechanisms of the observed effects of tissue-specific *Argonaute* genes’ knockdown on the radioresistance of *Drosophila*. γ-Irradiation caused the activation of genes provided the response to genotoxic stress, in particular, its coordination, nucleotide excision repair, and repair of double-strand breaks by non-homologous end joining (*Gadd45, Xpc, Ku80*), as well as the response to proteotoxic stress (*Hsp68*). These genes belong to the basic signaling pathways of reaction to acute irradiation, and their activation ensures survival under adverse conditions [80,84]. Indeed, in vivo and in vitro studies established that the activity of DNA damage recognition and repair genes is necessary for the normal reaction of cells and an organism to the action of ionizing radiation, the formation of specific responses [36,37,40,41]. Similarly, genes encoding heat shock proteins are activated under acute radiation and provide an adaptive response to stress [38,39,85]. We also expected changes in the expression of genes of autophagy and ER stress response—since, in studies on rodent and human cell lines, a connection between these mechanisms and the response to radiation has been established [86,87,88,89,90]. However, their changes in our study were small and inconsistent. On the other hand, it is important to note that we observed pronounced changes in the expression of genes of response to genotoxic and proteotoxic stress both in variants with RNA interference of the *Argonaute* genes and without activation of RNA interference.

It should be noted that in meta-analysis and bioinformatics studies of the radiosensitivity of normal and tumor cells, it was shown that genes of DNA damage response and repair, as well as genes of antioxidant defense, play a key role in the reaction of healthy tissues to the action of ionizing radiation [40]. In our study on the *Drosophila* model, it was shown that RNA interference of *Argonaute* genes increases the activity of orthologs of at least some genes important for this reaction (in particular, *GADD45A, XPC, XRCC3*). At the same time, a normal cellular response is maintained under in vivo irradiation conditions. However, these changes do not always provide benefits for the survival of the whole organism under radiation conditions. Suppression of the activity of some *Argonaute* genes could be a possible strategy for increasing the stress resistance of healthy tissues of higher organisms, including humans, but additional detailed studies are required to assess this perspective.

Irradiation increased the activity of retrotransposons in experimental variants without activation of RNA interference of the *Argonaute* genes. Indeed, it is known that damaging environmental factors (including ionizing radiation) can disrupt epigenetic control, and as a consequence, cause the activation of transposable elements. An increase in their activity is characterized by early manifestation and persistence, which makes it possible to use transposons as biomarkers of exposure to environmental stressors [91,92,93]. Surprisingly, irradiated *Drosophila* with *Argonaute* genes’ knockdown had lower levels of retrotransposon expression than irradiated animals without knockdown. One of the reasons may be associated with the complex interaction of the different factors involved in transposon activity regulation. As transposition activation factors, radiation-induced disruption of DNA integrity can act, which interacts with radiation-induced blockage of the transcriptional apparatus and epigenetic regulation of retrotransposition process in tissue-specific manner. It seems to be that the final level of transposon activity is determined by the balance of these factors. It should be noted that in this part of the experiment, one biological replication was carried out, and further verification is necessary to confirm the obtained data.

Additionally, it should be noted that we observed a greater sensitivity to irradiation in males than in females. This may be due to the specificity of the epigenome in different sexes. For example, males have more heterochromatic DNA than females, due to the presence of a Y chromosome with a large number of repeats [94]. Consequently, they are more sensitive to changes in the functioning of systems that regulate gene expression and repression of transposable elements.

## 4. Materials and Methods

### 4.1. Drosophila Melanogaster Strains and Induction of Argonaute Genes’ Knockdown

The wild-type *Canton-S* strain was used to assess age-related changes in gene expression.

In experiments to study the influence of tissue-specific knockdown of genes of the *Argonaute* family (*AGO1, AGO2, AGO3, piwi*) on the lifespan and the effects of γ-irradiation, the tested flies were obtained based on the GAL4/UAS system [95,96,97]. We used strains carrying double-stranded RNA (dsRNA) for RNA interference of these genes under the control of the *UAS* promoter (*RNAi-AGO1, RNAi-AGO2, RNAi-AGO3, RNAi-piwi*, respectively) and strains expressing the conditional (mifepristone-inducible) driver GAL4-GeneSwitch in specific tissues (*GS-elav*-in the nervous system, *GS-S106*-in the fat body, *GS-TIGS-2*-in the digestive system, *GS-Mhc*-in the muscles) (see Table S9).

The use of GAL4-GeneSwitch controls the expression level of a studied gene, the stage of development (imago), and the age of flies, at which the expression is induced, as well as the localization of suppression of the studied genes (ubiquitous or tissue-specific). First, the choice of tissue-specific expression was associated with the topicality of studying the role of *Argonaute* genes in various tissues in regulating lifespan and aging. Second, ubiquitous RNA interference in itself reduces the lifespan of *Drosophila*, while tissue-specific (including the drivers that were used in our study) does not have a negative effect on the lifespan [98]. In addition, the use of conditional GAL4 excludes the influence on the lifespan of an unequal genetic background in experimental and control animals.

To obtain experimental flies of each of the genotypes, virgin females of a line with UAS-construction and males of a line with GAL4-GeneSwitch were crossed. In males and virgin females obtained by crossing, an *Argonaute* gene knockdown was induced by mifepristone (RU486, Sigma-Aldrich, St. Louis, MO, USA) at a concentration of 3.2 mg/mL in ethanol, which was dripped onto a nutrient medium at 30 μL [99]. Control variants were obtained by the same crosses, but were kept in the medium without mifepristone. The decrease in the activity of *Argonaute* genes was verified by using RT-PCR analysis (Figure S4).

### 4.2. Lifespan Assay

Flies were kept at 25 C, 12:12 day-night regimen in climate chamber Binder KBF720-ICH (Binder, Tuttlingen, Germany) on nutrient medium (gram per 1 L): agar agar-5.2, dry yeast-32.1, glucose-136.9, yellow cornmeal-92.0 [100]. To prevent simple fungus and bacteria growth, a 10% solution of methyl 4-hydroxybenzoate (Sigma-Aldrich, St. Louis, MO, USA) and a 50% solution of propionic acid (Sigma-Aldrich, St. Louis, MO, USA) were added.

To silence the target genes, females expressing dsRNA under control of UAS sequences were crossed with GAL4 driver males. The F1 males and virgin females were used. Experimental flies were sorted by sex using CO_2_ anesthesia and were kept separately, 30 animals per *Drosophila* vial (Genesee Scientific, San Diego, CA, USA) with 5 mL of nutrient medium (see above) and 30 μL mifepristone solution, which was applied to the surface of the nutrient medium [99]. Control F1 flies were maintained on medium without mifepristone (with 30 μL ethanol).

Flies were transferred to fresh medium without anesthesia twice a week. The number of dead flies was counted daily. Further lifespan parameters (particularly the mean and median lifespan, the age of 90% mortality, the mortality rate doubling time (MRDT)) were calculated. Experiments were done in one-two independent biological replicates (two replicates were used for flies with RNA interference of *Argonaute* genes in the nervous system and the fat body to confirm the positive lifespan effects).

Statistical analysis was carried out using nonparametric criteria. The survival curves were shaped using a Kaplan–Meier procedure. The comparative analysis of the shape of survival curves was made using the Kolmogorov–Smirnov test [101]. Both the Mantel–Cox [102] and Gehan–Breslow–Wilcoxon tests [103] were used to estimate the statistical differences in the median lifespan. A Wang–Allison test was used to estimate differences in the age of 90% mortality [104]. The statistical analyses of the data were carried out using STATISTICA software, version 6.1 (StatSoft, Tulsa, OK, USA) and R, version 2.15.1 (The R Foundation, Indianapolis, IN, USA).

### 4.3. Irradiation Conditions

Experimental and control flies were obtained and cultivated in the same manner as for the lifespan assay. At the age of 14 days, experimental flies were irradiated using a Cs-137 γ-source “Issledovatel” (Russia) with a dose rate of 0.74 Gy/min. The radiosensitivity of adult flies to acute γ-irradiation was measured in the preliminary tests. A number of previous experimental data demonstrated that *Drosophila* adult imagoes are highly resistant to ionizing radiation [39,105,106]. For example, fifty percent lethality 1 h postirradiation has been shown to be approximately 1228 Gy for adult males and 1250 Gy for adult females [106].

To determine the dose of γ-irradiation useful for the current study, male imagoes from the control groups (without induction of RNA interference) were irradiated at a dose of 200–1000 Gy with increments of 200 Gy (Figure S5). According to the survival rate measurements, the radiation dose of 700 Gy (dose between 600 Gy and 800 Gy) was selected for the following experiments. This dose can significantly reduce survival without an acute lethality effect. After the exposure to γ-radiation, experimental and control flies were kept under standard conditions on the medium without mifepristone. Next, their survival was assessed. Statistical analysis was similar to the lifespan assay.

To avoid possible small differences in the accumulated dose, flies of each of the studied variants were placed in vials with 30 individuals. A total of four vials per experimental variant were used, each of which can be considered as a biological replicate. Replicates’ data were statistically processed together.

### 4.4. Real-Time RT-PCR

The gene expression analyses were carried out using whole *Drosophila* bodies or their parts (heads, thoraxes, or abdomens). In the case of whole flies, 10 males or 10 females were prepared per variant of the experiment. In other cases, 30 males or 30 females were partitioned into heads, thoraxes, or abdomens, places to separate tubes, and used for further procedures.

RNA was isolated by Aurum Total RNA mini kit (Bio-Rad, Hercules, CA, USA). To determine total RNA concentration was used Quant-iT RNA Assay Kit (Life Technologies, Eugene, OR, USA). Reverse transcription was performed using the iScript cDNA Synthesis Kit (Bio-Rad, USA). The mix for RT-PCR was prepared by iTaq Universal SYBR Green Supermix (Bio-Rad, USA) with primers listed in Table S10. The reaction was carried out on the CFX96 Real-Time PCR Detection System (Bio-Rad, USA) using the following parameters: One cycle of 95 °C for 30 s; 40 cycles of 95 °C for 10 s and 60 °C for 30 s. Expression levels of target genes were calculated relative to the expression of reference genes (*β-Tubulin, RpL32, EF1α*) using the CFX Manager 3.1 software (Bio-Rad, USA) by the 2^−∆∆Ct^ method [107]. The ∆∆C_t_ value was calculated as ∆C_t_ (Experimental sample) − ∆C_t_ (Control sample), and each value of ∆C_t_ = C_t_ (Target gene) − C_t_ (Reference genes), where C_t_—cycle thresholds. Experiments were carried out in two independent biological replicates, with three technical replicates in each.

RNA and cDNA samples were prepared using the equipment of the Molecular Biology Core Facility (IB FRC Komi SC UB RAS, Syktyvkar, Russia).

## 5. Conclusions

For the first time, we investigated the role of genes of the *Argonaute* family in regulating lifespan and radioresistance at the level of a whole complex organism using the in vivo model of *Drosophila melanogaster*. We found that a tissue-specific decrease in the activity of genes of the *Argonaute* family causes changes in lifespan and resistance to γ-irradiation at a dose of 700 Gy, depending on the gene and tissue in which a gene knockdown was triggered. In most cases, these parameters were reduced or did not change significantly in flies with tissue-specific RNA interference. Surprisingly, *piwi* knockdown in both the fat body and the nervous system, as well as *AGO1* and *AGO3* RNA interference in some cases caused a lifespan increase. Such positive changes were associated with increased expression of some stress response genes, but apparently, did not depend on the activity of transposons. At the same time, changes in radioresistance depended on the tissue in which the gene was knocked out. Thus, neuronal RNA interference of the *Argonaute* genes predominantly reduced the survival of irradiated flies, while RNA interference in the fat body increased the radioresistance of females.

The mechanism of epigenetic control using small RNAs is highly evolutionary conserved and persists through animal phylogeny [13]. Accordingly, in vivo studies in animal models (such as fruit flies or nematodes) suggest the function of small RNA orthologs, as well as proteins of their biogenesis in other animals, including humans. At the same time, epigenetic mechanisms are highly susceptible to external stimuli and affect a wide range of cellular processes, and small RNA biogenesis genes and proteins can be targets for potential geroprotectors and drugs in age-related diseases [1,2]. Indeed, it was found that the dysregulation of their activity is associated with the development of a number of age-related diseases, including cancer, inflammatory, neurodegenerative, cardiovascular, metabolic, and immune disorders [108,109,110,111,112,113,114,115,116]. We have found that suppression of some genes of the *Argonaute* family can prolong the life of fruit flies or enhance their radioresistance. This indicates the potential for their use as targets for geroprotective or radioprotective interventions (for example, using selective pharmacological drugs). However, a detailed study of the molecular mechanisms associated with the observed effects and possible negative consequences affecting quality of life is required.

## Data Availability

The data presented in this study are available on request from the corresponding author.

## Supplementary Materials

The following are available online at https://www.mdpi.com/1422-0067/22/5/2396/s1. Figure S1: Age-related changes in the expression of *Argonaute* genes in heads, thoraxes, abdomens of wild-type *Canton-S* males and females. Figure S2: Age-related changes in the expression of transposable elements in heads, thoraxes, abdomens of wild-type *Canton-S* males and females. Figure S3: Age-related changes in the expression of stress response genes in heads, thoraxes, abdominals of wild-type *Canton-S* males and females. Figure S4: Knockdown of *AGO1, AGO2, AGO3,* and *piwi* in investigated flies. Figure S5: Effects of acute γ-irradiation on the survival of *Drosophila* male imago from the controls for RNAi of *AGO1, AGO2, AGO3, piwi* in the fat body and nervous system. Table S1: Lifespan parameters of flies with tissue-specific knockdown of the *Argonaute* genes. Table S2: Survival of flies with tissue-specific knockdown of the *Argonaute* genes in the condition of γ-irradiation. Table S3: Age-related changes of gene expression in different parts of wild-type Canton-S males. Table S4: Age-related changes of gene expression in different parts of wild-type Canton-S females. Table S5: Mean relative gene expression of flies with tissue-specific AGO1 knockdown in the condition of γ-irradiation. Table S6: Mean relative gene expression of flies with tissue-specific AGO2 knockdown in the condition of γ-irradiation. Table S7: Mean relative gene expression of flies with tissue-specific AGO3 knockdown in the condition of γ-irradiation. Table S8: Mean relative gene expression of flies with tissue-specific piwi knockdown in the condition of γ-irradiation. Table S9: *Drosophila melanogaster* strains. Table S10: Primers for real-time PCR.
