# Supplementary material for: Tissue-Specific Knockdown of Genes of the Argonaute Family Modulates Lifespan and Radioresistance in Drosophila melanogaster
Source: Int J Mol Sci. 2021 Feb 27;22(5):2396. doi: 10.3390/ijms22052396 (PMC7957547; doi:10.3390/ijms22052396)
Supplement: Supplementary file 1 [file ijms-22-02396-s001.pdf]

Tissue-Specific Knockdown of Genes of the *Argonaute* Family Modulates Lifespan and Radioresistance in *Drosophila Melanogaster*

Ekaterina Proshkina, Elena Yushkova, Liubov Koval, Nadezhda Zemskaya, Evgeniya Shchegoleva, Ilya Solovev, Daria Yakovleva, Natalya Pakshina, Natalia Ulyasheva, Mikhail Shaposhnikov, Alexey Moskalev

Supplementary Materials

Table S1. Lifespan parameters of flies with tissue-specific knockdown of the *Argonaute* genes.

Genotype	Sex	Replicate	RU486	$\bar{X}$±SE	M	90%	MRDT	N
GS-elav>RNAi-AGO1	Males	1	-	62.0±1.0	61	76	8.41	146
			+	67.6±1.0 **	68 ***	79	6.60	149
		2	-	52.6±0.6	54.5	63	5.16	190
			+	50.4±0.7 *	52 *	62	5.46	171
	Females	1	-	73.0±1.4	74	88	7.38	111
			+	71.0±2.3	78	91	9.56	98
		2	-	62.5±1.6	66	74	5.83	83
			+	59.2±1.2 ***	63 ***	69 ***	5.06	119
GS-S106>RNAi-AGO1	Males	1	-	57.6±1.0	56	72	8.17	144
			+	59.5±1.0	61	72	6.66	131
	Females	1	-	57.3±2.8	71	89	20.4	129
			+	72.0±1.9	76 **	91	8.94	121
		2	-	65.1±0.8	67	74	4.57	197
			+	63.7±0.9	65 **	77 *	5.78	177
GS-TIGS-2>RNAi-AGO1	Males	1	-	52.3±1.0	51	65	7.25	141
			+	51.2±0.9	49.5	68	7.20	150
	Females	1	-	66.3±2.3	76	91	12.29	122
			+	64.2±2.3	74	92	13.34	140
GS-Mhc>RNAi-AGO1	Males	1	-	47.9±1.0	49	60	7.04	134
			+	29.5±1.4 ***	32 ***	49 ***	12.56	138
	Females	1	-	55.7±1.4	59	72	7.55	115
			+	24.9±1.9 ***	11 ***	53 ***	36.91	107
GS-elav>RNAi-AGO2	Males	1	-	45.1±1.1	44	61	9.02	153
			+	42.7±0.9 **	42 *	56 *	6.99	146
		2	-	52.9±0.9	55	65	6.80	180
			+	53.3±0.7	55	63 ***	5.00	172
	Females	1	-	69.5±1.1	73	84	6.28	138
			+	61.2±1.6 ***	63 ***	80	9.13	139
		2	-	64.4±1.1	68	77	5.97	187
			+	60.9±0.9 ***	64 ***	75	6.60	174
GS-S106>RNAi-AGO2	Males	1	-	40.0±1.2	42	57	9.55	158
			+	40.0±1.5	41	60	10.83	115
		2	-	58.1±0.8	58	70	6.31	173
			+	57.2±0.6	57	64 ***	5.21	171
	Females	1	-	63.9±1.7	69	83	10.26	164
			+	66.5±1.3	66	84	7.66	151
		2	-	64.1±0.8	65	78	6.41	192
			+	63.8±0.9	65	77	5.57	170
GS-TIGS-2>RNAi-AGO2	Males	1	-	46.5±0.9	45	60	9.61	159

			+	46.5±1.1	45	64	8.66	122
	Females	1	-	80.1±1.1	83	92	5.53	147
			+	76.2±1.2 ***	78 ***	88 *	5.86	136
GS-Mhc>RNAi-AGO2	Males	1	-	41.7±1.1	39	57	9.17	136
			+	27.0±0.9 ***	29 ***	37 ***	8.40	123
	Females	1	-	74.7±1.5	75	84	4.63	66
			+	14.9±1.7 ***	8 ***	43 ***	6931.47	83
GS-elav>RNAi-AGO3	Males	1	-	59.9±0.9	62	70	5.98	136
			+	52.5±1.3 ***	56 ***	64 ***	6.26	125
		2	-	51.3±1.6	54	64	7.23	66
			+	54.1±1.0	53	64	5.92	79
	Females	1	-	66.5±2.1	77	84	9.32	125
			+	69.3±1.1 *	74 *	83	6.86	148
		2	-	61.8±2.2	70	83	10.22	98
			+	69.5±1.5	74	82	6.34	87
GS-S106>RNAi-AGO3	Males	1	-	57.0±0.8	57	68	5.67	128
			+	56.9±0.9	57	67	5.96	152
		2	-	54.5±0.9	56	68	6.68	169
			+	54.3±1.0	56	66	7.10	159
	Females	1	-	77.6±1.2	77.5	93	7.84	102
			+	71.0±1.9 ***	77	86 ***	7.59	109
		2	-	67.8±1.2	71	84	7.54	220
			+	68.5±1.0	71	81 ***	6.39	220
GS-TIGS-2>RNAi-AGO3	Males	1	-	51.8±0.9	53	59	5.45	129
			+	46.5±0.9 ***	50 ***	54	6.50	132
	Females	1	-	57.1±2.7	70.5	86	16.10	112
			+	62.1±2.3	73	87	12.80	126
GS-Mhc>RNAi-AGO3	Males	1	-	57.5±0.6	59	64	3.93	139
			+	26.2±0.5 ***	25 ***	36 ***	4.24	142
	Females	1	-	74.0±1.7	80.5	89	7.75	112
			+	16.4±1.1 ***	12 ***	33 ***	13.73	86
GS-elav>RNAi-piwi	Males	1	-	62.5±1.1	64	78	7.42	154
			+	69.5±0.9 ***	72 ***	82 ***	6.16	137
	Females	1	-	72.8±2.0	75	85	6.27	73
			+	76.9±2.1 ***	82 ***	88	5.46	64
		2	-	68.6±1.8	69.5	79	5.84	36
			+	72.1±1.3	71	81	4.24	41
GS-S106>RNAi-piwi	Males	1	-	61.7±1.0	61	75	7.50	119
			+	62.6±1.3 *	64	77	7.82	119
		2	-	53.2±0.7	53	64	5.42	159
			+	55.1±1.0 *	57 **	64	5.01	128
	Females	1	-	62.9±2.1	71	84	11.00	114
			+	65.1±1.9	69	84	9.12	118
		2	-	63.6±1.1	66	78	6.93	168
			+	64.2±1.5	66	80	7.68	147
GS-TIGS-2>RNAi-piwi	Males	1	-	60.7±0.9	64	71	5.94	162
			+	59.4±0.8	59	73	6.79	170
	Females	1	-	77.8±0.9	80.5	86	5.24	138
			+	76.6±1.0	77	85	5.07	131
GS-Mhc>RNAi-piwi	Males	1	-	53.4±1.0	55.5	62	4.36	86
			+	26.9±1.6 ***	26 ***	51 ***	15.56	93
	Females	1	-	69.1±4.6	82	85	8.76	115
			+	16.5±2.3 ***	10 ***	37 ***	21.94	126

$\bar{X}$±SE - mean lifespan (days)

M - median lifespan (days)

90% - the age of 90 % mortality (days)
MRDT - the mortality rate doubling time (days)
N - the number of flies in a sample
* - differences between variants with *Argonautes*' knockdown induction with RU486 and without knockdown are statistically significant with $p < 0.05$ (fifth column - Mantel-Cox test, sixth column - Gehan-Breslow-Wilcoxon test, seventh column - Wang-Allison test)
** - $p < 0.01$
*** - $p < 0.001$

Table S2. Survival of flies with tissue-specific knockdown of the *Argonaute* genes in the condition of γ-irradiation.

Genotype	Sex	RU486	IR	X̄±SE	M	90%	MRDT	N
GS-elav>RNAi-AGO1	Males	-	0	47.5±1.4	49.5	64	8.42	118
		+	0	47.4±1.4	51	66	8.60	124
		-	700	21.4±0.5	20	27	3.15	122
		+	700	20.0±0.5	21	27	3.53	113
	Females	-	0	58.4±1.6	66	80	9.29	163
		+	0	54.1±1.3 ***	59 ***	68 ***	7.10	116
		-	700	18.1±0.6	17	21	8.74	109
		+	700	29.4±1.2 ***	21 ***	47 ***	9.81	106
GS-S106>RNAi-AGO1	Males	-	0	42.2±1.2	44	59	8.20	118
		+	0	43.4±1.2	41	60	8.42	115
		-	700	19.5±0.6	18	28	4.29	116
		+	700	26.6±0.7 ***	28 ***	34 ***	4.28	117
	Females	-	0	60.5±1.3	66	75	7.57	188
		+	0	57.9±1.4 ***	66 **	70 ***	6.11	114
		-	700	19.4±0.9	18	31	10.60	118
		+	700	31.4±1.5 ***	28 ***	56 ***	13.79	111
GS-elav>RNAi-AGO2	Males	-	0	50.1±1.1	52	60	6.17	115
		+	0	54.6±0.8	56 **	65	4.76	116
		-	700	30.1±0.9	31	42	6.15	119
		+	700	22.0±1.0 ***	17 ***	38	11.77	118
	Females	-	0	60.3±1.3	63	73	5.86	114
		+	0	60.7±1.4 *	67 **	73	5.87	118
		-	700	29.4±1.4	21	51	13.09	101
		+	700	26.3±1.5	17 ***	56	22.70	110
GS-S106>RNAi-AGO2	Males	-	0	43.1±1.7	46	63	11.01	122
		+	0	44.9±1.1 *	46	58 **	6.46	107
		-	700	35.5±0.8	38	44	4.54	121
		+	700	32.8±0.8 **	34 **	41	4.94	118
	Females	-	0	63.9±1.1	66	76	6.12	140
		+	0	65.1±1.3	69	75	5.46	98
		-	700	25.6±1.3	20	46	14.58	115
		+	700	27.9±1.6 *	17	59 ***	23.86	128
GS-elav>RNAi-AGO3	Males	-	0	50.4±1.2	52	67	7.27	116
		+	0	48.4±1.4	51	63 *	7.02	99
		-	700	27.9±0.8	28	38	5.78	118
		+	700	19.1±0.7 ***	17 ***	30 **	6.58	117
	Females	-	0	67.2±1.6	73	83	7.45	132
		+	0	67.2±1.6	70	81	6.90	110
		-	700	20.8±1.0	17	41	15.15	114
		+	700	23.3±1.1	17	45	15.38	114
GS-S106>RNAi-AGO3	Males	-	0	49.3±1.4	51	63	8.15	115
		+	0	50.7±1.4	51.5	66	8.03	112
		-	700	28.1±0.9	30	38	6.30	121
		+	700	25.7±0.9	25	38	7.92	116
	Females	-	0	67.3±1.7	74	83	7.26	125
		+	0	67.6±1.5	70	83	7.52	121
		-	700	28.9±1.5	27	51	15.23	109
		+	700	38.4±1.6 ***	41 ***	60 ***	11.88	114
GS-elav>RNAi-piwi	Males	-	0	45.7±1.2	46	60	6.98	113
		+	0	46.0±1.2	48	60	6.70	95
		-	700	24.0±0.7	25	34	5.80	124
		+	700	17.2±0.5 ***	14 ***	26 ***	5.55	118

	Females	-	0	63.3±1 .0	66	74	5.80	129
		+	0	66.3±1.4 ***	68 ***	80 **	7.22	127
		-	700	35.2±1.0	38	51	6.56	114
		+	700	22.1±1.0 ***	17 ***	40	12.82	115
GS-S106>RNAi-piwi	Males	-	0	48.9±1.3	51	63	6.98	97
		+	0	53.9±1.1 ***	59 **	66 **	5.86	113
		-	700	22.1±0.8	25	31	5.31	103
		+	700	25.0±0.8 ***	25 **	38 ***	6.34	120
	Females	-	0	64.3±1.4	68	80	7.24	129
		+	0	60.2±1.8	68	77	9.49	147
		-	700	23.9±1.4	17	51	24.45	116
		+	700	46.6±1.5 ***	51 ***	63 ***	8.34	114

RU486 – mifepristone treatment

IR – ionizing irradiation dose (Gy)

$\bar{X}$±SE - mean lifespan (days)

M - median lifespan (days)

90% - the age of 90 % mortality (days)

MRDT - the mortality rate doubling time (days)

N - the number of flies in a sample

* - differences between variants with *Argonautes*' knockdown induction with RU486 and without knockdown are statistically significant with $p < 0.05$ (fifth column - Mantel-Cox test, sixth column - Gehan-Breslow-Wilcoxon test, seventh column - Wang-Allison test)

** - $p < 0.01$

*** - $p < 0.001$

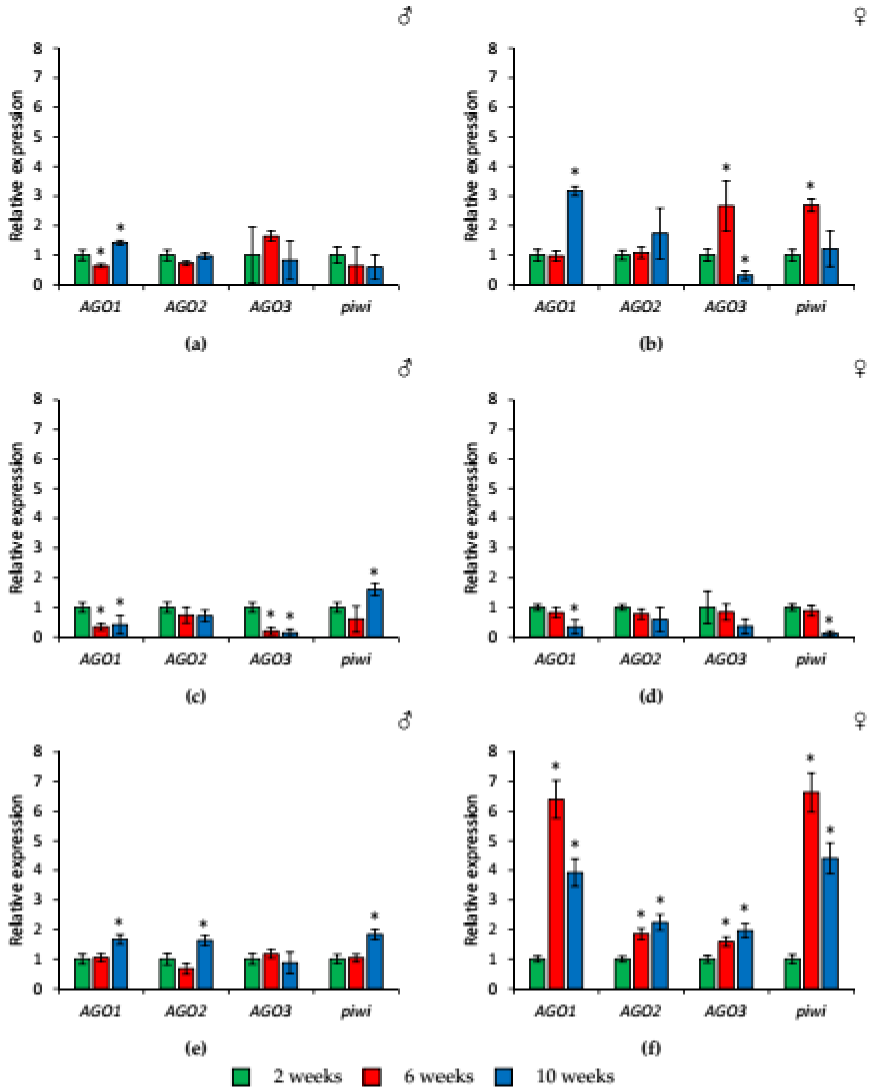

Figure S1. Age-related changes in the expression of *Argonaute* genes in heads (**a, b**), toraxes (**c, d**), abdomens (**e, f**) of wild-type *Canton-S* males (**a, c, e**) and females (**b, d, f**). Differences between relative expression levels of the investigated genes at the age of 2 weeks and at the ages of 6 and 10 weeks are statistically significant with * - $p < 0.05$ (Mann-Whitney U-test).

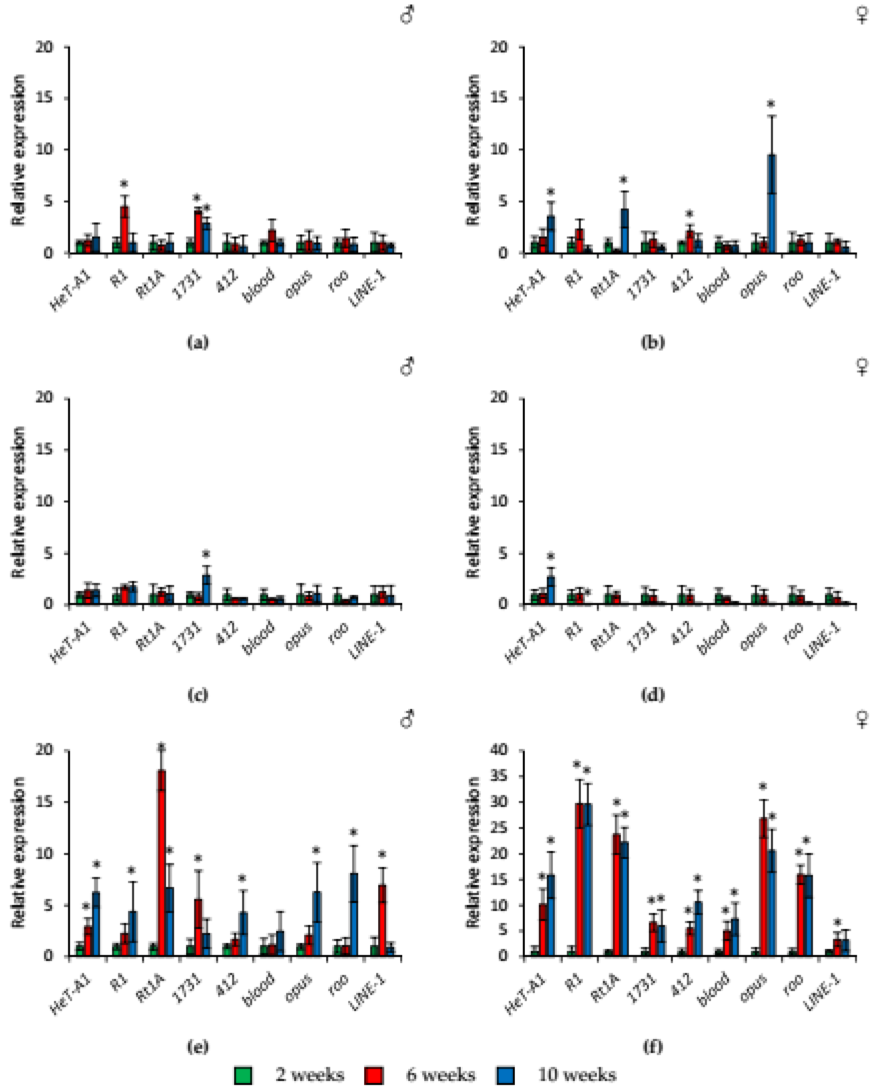

Figure S2. Age-related changes in the expression of retrotrasposons in heads (**a, b**), toraxes (**c, d**), abdomens (**e, f**) of wild-type *Canton-S* males (**a, c, e**) and females (**b, d, f**). Differences between relative expression levels of the investigated genes at the age of 2 weeks and at the ages of 6 and 10 weeks are statistically significant with * - p < 0.05 (Mann-Whitney U-test).

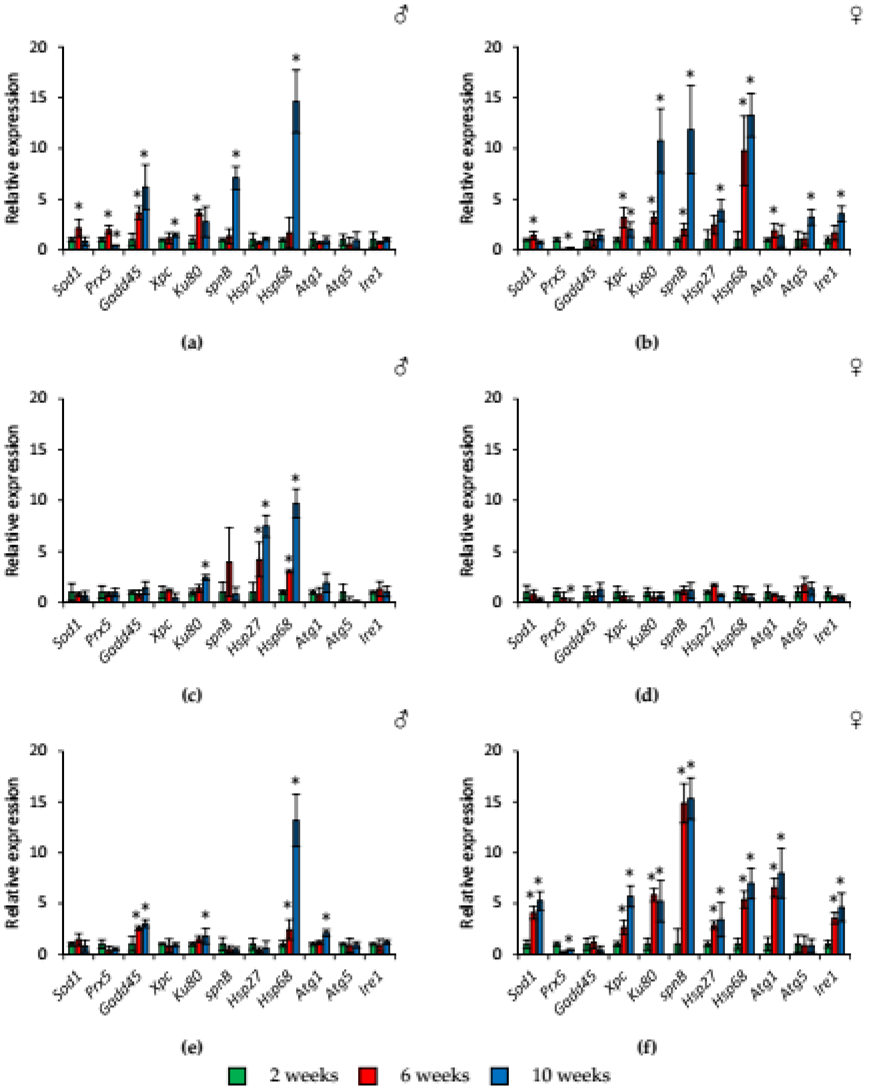

Figure S3. Age-related changes in the expression of stress response genes in heads (**a, b**), toraxes (**c, d**), abdomens (**e, f**) of wild-type *Canton-S* males (**a, c, e**) and females (**b, d, f**). Differences between relative expression levels of the investigated genes at the age of 2 weeks and at the ages of 6 and 10 weeks are statistically significant with * - $p < 0.05$ (Mann-Whitney U-test).

Table S3. Age-related changes of gene expression in different parts of wild-type *Canton-S* males.

Gene	Replicate	Whole body			Heads			Toraxes			Abdomens		
		2 weeks	6 weeks	10 weeks	2 weeks	6 weeks	10 weeks	2 weeks	6 weeks	10 weeks	2 weeks	6 weeks	10 weeks
AGO1	1	1.00± 0.08	1.15± 0.08	1.67± 0.08	1.00± 0.06	0.72± 0.05	1.53± 0.12	1.00± 0.10	0.40± 0.08	0.48± 0.09	1.00± 0.10	1.24± 0.12	1.85± 0.11
	2	1.00± 0.09	0.53± 0.11	1.49± 0.13	1.00± 0.03	0.64± 0.03	1.29± 0.13	1.00± 0.05	0.19± 0.08	0.29± 0.12	1.00± 0.09	0.93± 0.10	1.48± 0.15
AGO2	1	1.00± 0.08	1.28± 0.07	1.80± 0.13	1.00± 0.09	0.86± 0.05	0.89± 0.07	1.00± 0.07	0.89± 0.15	0.90± 0.15	1.00± 0.08	0.87± 0.06	1.49± 0.12
	2	1.00± 0.07	0.60± 0.14	1.40± 0.13	1.00± 0.10	0.73± 0.13	1.02± 0.14	1.00± 0.10	0.54± 0.08	0.50± 0.14	1.00± 0.10	0.55± 0.11	1.81± 0.10
AGO3	1	1.00± 0.11	0.39± 0.07	2.33± 0.15	1.00± 0.11	1.45± 0.12	1.38± 0.16	1.00± 0.12	0.25± 0.16	0.23± 0.12	1.00± 0.09	1.26± 0.08	1.19± 0.10
	2	1.00± 0.01	0.56± 0.10	1.26± 0.15	1.00± 0.08	1.81± 0.13	0.45± 0.09	1.00± 0.09	0.14± 0.07	0.06± 0.12	1.00± 0.09	1.10± 0.08	0.61± 0.09
piwi	1	1.00± 0.09	1.07± 0.13	2.86± 0.12	1.00± 0.08	1.20± 0.13	0.78± 0.13	1.00± 0.07	1.02± 0.13	1.42± 0.10	1.00± 0.06	1.23± 0.16	1.58± 0.13
	2	1.00± 0.08	0.55± 0.03	1.96± 0.10	1.00± 0.09	0.25± 0.11	0.29± 0.07	1.00± 0.10	0.34± 0.06	1.81± 0.08	1.00± 0.04	0.93± 0.07	2.07± 0.18
HeT-A1	1	1.00± 0.06	1.16± 0.11	9.26± 0.20	1.00± 0.10	1.36± 0.11	1.94± 0.11	1.00± 0.09	1.73± 0.10	1.81± 0.13	1.00± 0.07	3.62± 0.20	8.45± 0.10
	2	1.00± 0.05	0.62± 0.10	15.19± 0.23	1.00± 0.10	1.07± 0.13	1.11± 0.13	1.00± 0.07	1.14± 0.09	1.11± 0.13	1.00± 0.08	2.19± 0.09	4.12± 0.14
R1-element	1	1.00± 0.08	1.59± 0.08	11.27± 0.30	1.00± 0.09	5.85± 0.20	0.55± 0.14	1.00± 0.07	1.85± 0.14	1.99± 0.13	1.00± 0.06	3.43± 0.20	6.22± 0.12
	2	1.00± 0.09	1.09± 0.08	6.31± 0.25	1.00± 0.08	3.35± 0.18	1.55± 0.14	1.00± 0.08	1.54± 0.14	1.59± 0.09	1.00± 0.06	1.43± 0.10	2.22± 0.11
Rt1a	1	1.00± 0.06	2.33± 0.10	5.45± 0.19	1.00± 0.09	1.18± 0.12	1.61± 0.09	1.00± 0.09	1.45± 0.10	1.46± 0.09	1.00± 0.07	21.55± 0.37	3.67± 0.10
	2	1.00± 0.07	1.29± 0.14	1.42± 0.05	1.00± 0.07	0.35± 0.09	0.49± 0.11	1.00± 0.06	1.21± 0.15	0.81± 0.10	1.00± 0.10	15.57± 0.21	9.99± 0.17
1731	1	1.00± 0.07	7.15± 0.14	6.89± 0.18	1.00± 0.10	4.64± 0.20	1.93± 0.07	1.00± 0.07	1.15± 0.12	3.58± 0.16	1.00± 0.11	7.31± 0.16	1.47± 0.12
	2	1.00± 0.08	1.95± 0.09	2.89± 0.18	1.00± 0.06	3.40± 0.20	3.70± 0.19	1.00± 0.07	0.61± 0.11	2.05± 0.09	1.00± 0.12	3.15± 0.17	3.51± 0.15
412	1	1.00± 0.09	1.20± 0.15	13.48± 0.23	1.00± 0.07	1.19± 0.14	1.23± 0.08	1.00± 0.10	0.76± 0.11	0.69± 0.06	1.00± 0.08	2.19± 0.08	6.23± 0.17
	2	1.00± 0.06	0.17± 0.02	5.43± 0.25	1.00± 0.09	0.43± 0.11	0.14± 0.09	1.00± 0.10	0.56± 0.11	0.57± 0.12	1.00± 0.08	1.14± 0.07	2.37± 0.13
blood	1	1.00± 0.05	0.61± 0.13	4.47± 0.15	1.00± 0.10	3.04± 0.16	0.76± 0.07	1.00± 0.07	0.66± 0.13	0.76± 0.06	1.00± 0.13	1.53± 0.12	3.12± 0.11
	2	1.00± 0.12	0.12± 0.08	1.85± 0.10	1.00± 0.11	1.39± 0.12	1.36± 0.08	1.00± 0.06	0.41± 0.07	0.52± 0.15	1.00± 0.08	0.80± 0.10	1.84± 0.08
opus	1	1.00± 0.09	1.29± 0.15	7.05± 0.09	1.00± 0.08	1.80± 0.13	1.30± 0.07	1.00± 0.06	1.07± 0.10	0.93± 0.12	1.00± 0.09	1.80± 0.10	9.24± 0.25
	2	1.00± 0.10	0.76± 0.07	4.21± 0.16	1.00± 0.01	0.80± 0.13	0.70± 0.08	1.00± 0.07	0.70± 0.11	1.32± 0.14	1.00± 0.09	2.40± 0.12	3.77± 0.15
roo	1	1.00± 0.08	0.50± 0.13	8.90± 0.18	1.00± 0.08	1.55± 0.08	0.78± 0.10	1.00± 0.08	0.66± 0.09	0.59± 0.08	1.00± 0.09	1.48± 0.15	11.92± 0.30
	2	1.00± 0.08	0.27± 0.13	3.37± 0.09	1.00± 0.04	1.28± 0.10	1.16± 0.08	1.00± 0.11	0.24± 0.12	1.06± 0.10	1.00± 0.07	0.55± 0.06	4.13± 0.21
microsatellite/ LINE-1	1	1.00± 0.07	0.86± 0.07	3.08± 0.17	1.00± 0.09	0.80± 0.13	0.50± 0.03	1.00± 0.09	1.46± 0.15	0.56± 0.08	1.00± 0.06	9.47± 0.24	1.40± 0.12
	2	1.00±	0.62±	1.97±	1.00±	1.22±	0.89±	1.00±	1.04±	1.32±	1.00±	4.71±	0.61±

		0.06	0.07	0.16	0.09	0.09	0.10	0.08	0.07	0.08	0.06	0.26	0.08
Sod1	1	1.00± 0.02	0.62± 0.06	0.79± 0.10	1.00± 0.09	3.26± 0.18	1.59± 0.17	1.00± 0.08	1.03± 0.07	0.80± 0.08	1.00± 0.07	2.03± 0.15	0.94± 0.07
	2	1.00± 0.11	0.46± 0.03	0.34± 0.12	1.00± 0.08	1.14± 0.10	0.08± 0.02	1.00± 0.06	0.53± 0.07	0.48± 0.07	1.00± 0.07	0.81± 0.13	0.63± 0.08
Prx5	1	1.00± 0.10	0.23± 0.04	0.04± 0.02	1.00± 0.09	2.23± 0.14	0.28± 0.08	1.00± 0.05	0.58± 0.05	0.75± 0.14	1.00± 0.08	0.57± 0.07	0.74± 0.13
	2	1.00± 0.09	0.65± 0.08	0.09± 0.01	1.00± 0.10	1.61± 0.12	0.52± 0.05	1.00± 0.05	0.89± 0.15	1.23± 0.07	1.00± 0.10	0.18± 0.03	0.32± 0.06
Gadd45	1	1.00± 0.07	2.28± 0.16	2.36± 0.18	1.00± 0.11	4.30± 0.18	8.61± 0.23	1.00± 0.07	0.45± 0.06	1.21± 0.10	1.00± 0.09	2.85± 0.16	2.65± 0.14
	2	1.00± 0.06	4.85± 0.17	1.54± 0.19	1.00± 0.08	2.69± 0.12	3.97± 0.26	1.00± 0.08	1.25± 0.16	1.79± 0.12	1.00± 0.09	2.13± 0.16	3.54± 0.20
Xpc	1	1.00± 0.06	0.83± 0.13	1.62± 0.13	1.00± 0.06	1.10± 0.10	1.27± 0.17	1.00± 0.10	1.32± 0.17	1.04± 0.08	1.00± 0.09	0.94± 0.12	1.18± 0.12
	2	1.00± 0.08	1.16± 0.14	1.98± 0.10	1.00± 0.07	1.34± 0.10	1.65± 0.08	1.00± 0.11	1.02± 0.07	0.01± 0.03	1.00± 0.06	0.73± 0.05	0.68± 0.10
Ku80	1	1.00± 0.09	1.70± 0.10	1.69± 0.09	1.00± 0.07	4.28± 0.20	3.45± 0.09	1.00± 0.08	1.22± 0.12	2.20± 0.17	1.00± 0.11	1.92± 0.13	2.35± 0.15
	2	1.00± 0.10	1.32± 0.07	1.06± 0.10	1.00± 0.08	3.02± 0.13	1.87± 0.16	1.00± 0.09	1.52± 0.13	2.68± 0.11	1.00± 0.10	1.12± 0.13	1.22± 0.13
spn-B	1	1.00± 0.11	3.20± 0.19	0.46± 0.08	1.00± 0.09	1.76± 0.12	8.65± 0.25	1.00± 0.07	3.09± 0.13	0.57± 0.08	1.00± 0.11	0.35± 0.06	0.38± 0.05
	2	1.00± 0.10	1.59± 0.10	1.51± 0.07	1.00± 0.09	0.88± 0.06	5.54± 0.12	1.00± 0.07	4.73± 0.20	0.98± 0.06	1.00± 0.06	0.55± 0.15	0.57± 0.08
Hsp27	1	1.00± 0.09	1.20± 0.12	1.12± 0.11	1.00± 0.10	0.86± 0.07	0.96± 0.05	1.00± 0.08	5.91± 0.25	8.63± 0.13	1.00± 0.09	0.19± 0.04	0.20± 0.04
	2	1.00± 0.05	1.46± 0.07	1.59± 0.21	1.00± 0.08	0.42± 0.07	1.25± 0.17	1.00± 0.09	2.59± 0.11	6.33± 0.30	1.00± 0.08	0.71± 0.07	0.92± 0.10
Hsp68	1	1.00± 0.07	4.55± 0.25	4.25± 0.18	1.00± 0.06	2.48± 0.08	9.40± 0.34	1.00± 0.10	3.74± 0.15	12.34± 0.39	1.00± 0.08	3.27± 0.16	18.09± 0.30
	2	1.00± 0.06	1.98± 0.13	3.36± 0.10	1.00± 0.14	0.88± 0.11	20.40± 0.34	1.00± 0.05	2.52± 0.11	7.23± 0.15	1.00± 0.07	1.56± 0.06	8.72± 0.26
Atg1	1	1.00± 0.08	1.57± 0.08	1.08± 0.11	1.00± 0.10	0.96± 0.12	0.72± 0.14	1.00± 0.10	0.39± 0.09	2.21± 0.14	1.00± 0.09	1.49± 0.10	1.84± 0.10
	2	1.00± 0.08	1.24± 0.15	1.38± 0.11	1.00± 0.11	0.43± 0.06	1.23± 0.09	1.00± 0.10	0.99± 0.12	1.54± 0.14	1.00± 0.10	0.79± 0.10	2.39± 0.18
Atg5	1	1.00± 0.10	1.49± 0.07	0.44± 0.12	1.00± 0.08	0.21± 0.05	1.17± 0.10	1.00± 0.09	0.01± 0.01	0.03± 0.05	1.00± 0.06	1.26± 0.09	1.34± 0.16
	2	1.00± 0.11	1.03± 0.12	0.97± 0.04	1.00± 0.09	0.81± 0.05	0.70± 0.12	1.00± 0.07	0.23± 0.05	0.13± 0.05	1.00± 0.06	0.54± 0.07	0.58± 0.13
Ire1	1	1.00± 0.09	1.17± 0.06	1.39± 0.07	1.00± 0.08	0.80± 0.16	0.89± 0.17	1.00± 0.07	1.56± 0.16	1.24± 0.14	1.00± 0.09	0.69± 0.14	1.41± 0.12
	2	1.00± 0.07	0.75± 0.05	1.58± 0.14	1.00± 0.10	0.63± 0.08	1.25± 0.10	1.00± 0.09	1.02± 0.06	0.87± 0.06	1.00± 0.08	1.03± 0.16	1.02± 0.07

Table S4. Age-related changes of gene expression in different parts of wild-type *Canton-S* females.

Gene	Replicate	Whole body			Heads			Toraxes			Abdomens		
		2 weeks	6 weeks	10 weeks	2 weeks	6 weeks	10 weeks	2 weeks	6 weeks	10 weeks	2 weeks	6 weeks	10 weeks
AGO1	1	1.00± 0.06	1.37± 0.07	1.45± 0.06	1.00± 0.09	1.11± 0.09	3.68± 0.12	1.00± 0.04	1.02± 0.16	0.14± 0.19	1.00± 0.08	7.54± 0.17	2.82± 0.12
	2	1.00± 0.05	0.97± 0.07	1.95± 0.16	1.00± 0.08	0.86± 0.08	2.78± 0.11	1.00± 0.07	0.57± 0.09	0.54± 0.06	1.00± 0.07	5.11± 0.12	5.06± 0.17
AGO2	1	1.00± 0.10	1.28± 0.10	2.17± 0.09	1.00± 0.07	1.34± 0.12	2.29± 0.18	1.00± 0.06	0.93± 0.10	0.87± 0.10	1.00± 0.07	2.06± 0.17	2.01± 0.14
	2	1.00± 0.07	1.00± 0.09	1.63± 0.08	1.00± 0.08	0.83± 0.14	1.07± 0.09	1.00± 0.05	0.59± 0.13	0.29± 0.09	1.00± 0.10	1.73± 0.14	2.50± 0.19
AGO3	1	1.00± 0.07	1.68± 0.12	1.54± 0.07	1.00± 0.10	3.54± 0.08	0.53± 0.12	1.00± 0.06	1.10± 0.06	0.23± 0.15	1.00± 0.10	1.71± 0.05	2.15± 0.14
	2	1.00± 0.04	1.29± 0.13	1.22± 0.15	1.00± 0.05	1.36± 0.13	0.24± 0.13	1.00± 0.08	0.66± 0.10	0.50± 0.14	1.00± 0.09	1.43± 0.07	1.70± 0.15
piwi	1	1.00± 0.10	2.09± 0.17	1.67± 0.09	1.00± 0.06	2.93± 0.13	1.63± 0.12	1.00± 0.08	1.09± 0.15	0.14± 0.08	1.00± 0.07	7.43± 0.13	5.51± 0.20
	2	1.00± 0.09	1.62± 0.10	1.19± 0.14	1.00± 0.04	2.44± 0.19	0.78± 0.12	1.00± 0.10	0.68± 0.06	0.09± 0.05	1.00± 0.05	5.96± 0.26	3.61± 0.20
HeT-A1	1	1.00± 0.11	0.76± 0.06	2.52± 0.13	1.00± 0.09	1.28± 0.15	4.48± 0.30	1.00± 0.10	1.02± 0.16	3.36± 0.20	1.00± 0.10	5.23± 0.20	7.58± 0.19
	2	1.00± 0.09	1.35± 0.08	3.47± 0.12	1.00± 0.05	1.84± 0.08	2.80± 0.15	1.00± 0.09	1.35± 0.07	2.03± 0.14	1.00± 0.10	16.41± 0.22	22.86± 0.37
R1-element	1	1.00± 0.08	1.01± 0.14	2.06± 0.14	1.00± 0.07	1.44± 0.16	0.72± 0.14	1.00± 0.07	1.04± 0.12	0.01± 0.01	1.00± 0.08	17.12± 0.31	22.09± 0.26
	2	1.00± 0.09	0.61± 0.04	2.76± 0.24	1.00± 0.09	3.07± 0.09	0.12± 0.04	1.00± 0.07	1.50± 0.14	0.23± 0.03	1.00± 0.09	41.21± 0.20	36.02± 0.14
Rt1a	1	1.00± 0.09	0.35± 0.04	1.28± 0.18	1.00± 0.09	0.05± 0.03	1.29± 0.15	1.00± 0.06	0.51± 0.09	0.00± 0.03	1.00± 0.08	15.12± 0.29	14.69± 0.18
	2	1.00± 0.07	1.19± 0.07	3.78± 0.18	1.00± 0.08	0.47± 0.09	6.94± 0.26	1.00± 0.06	1.67± 0.14	0.35± 0.10	1.00± 0.08	32.15± 0.41	28.80± 0.28
1731	1	1.00± 0.06	0.97± 0.12	0.90± 0.11	1.00± 0.10	1.09± 0.13	0.45± 0.13	1.00± 0.10	0.59± 0.13	0.23± 0.05	1.00± 0.05	4.39± 0.21	4.63± 0.19
	2	1.00± 0.03	1.47± 0.12	1.58± 0.14	1.00± 0.12	1.54± 0.09	0.73± 0.13	1.00± 0.09	1.26± 0.08	0.05± 0.04	1.00± 0.06	8.75± 0.15	7.38± 0.17
412	1	1.00± 0.08	1.15± 0.11	1.77± 0.16	1.00± 0.10	1.61± 0.09	1.52± 0.10	1.00± 0.08	0.57± 0.08	0.46± 0.10	1.00± 0.06	3.24± 0.17	6.49± 0.16
	2	1.00± 0.11	1.32± 0.16	3.54± 0.28	1.00± 0.08	2.61± 0.09	1.02± 0.07	1.00± 0.10	1.39± 0.11	0.01± 0.03	1.00± 0.09	7.95± 0.15	14.95± 0.19
blood	1	1.00± 0.10	1.28± 0.11	2.15± 0.11	1.00± 0.04	0.51± 0.07	0.47± 0.14	1.00± 0.11	0.49± 0.06	0.41± 0.06	1.00± 0.09	2.89± 0.18	4.10± 0.19
	2	1.00± 0.09	0.93± 0.11	1.40± 0.15	1.00± 0.06	1.04± 0.16	0.82± 0.10	1.00± 0.08	0.86± 0.11	0.01± 0.01	1.00± 0.07	7.28± 0.20	10.93± 0.25
opus	1	1.00± 0.08	0.66± 0.07	1.50± 0.12	1.00± 0.09	0.83± 0.08	7.21± 0.17	1.00± 0.07	0.74± 0.06	0.01± 0.03	1.00± 0.12	15.71± 0.23	14.27± 0.15
	2	1.00± 0.06	1.36± 0.07	3.29± 0.15	1.00± 0.09	1.28± 0.14	12.06± 0.27	1.00± 0.07	1.25± 0.06	0.16± 0.05	1.00± 0.10	37.34± 0.16	27.27± 0.25
roo	1	1.00± 0.04	1.22± 0.13	1.27± 0.09	1.00± 0.09	1.03± 0.16	0.74± 0.14	1.00± 0.11	0.68± 0.13	0.35± 0.09	1.00± 0.09	14.16± 0.12	8.47± 0.14
	2	1.00± 0.08	1.87± 0.15	4.69± 0.23	1.00± 0.05	1.49± 0.15	1.36± 0.14	1.00± 0.10	1.02± 0.08	0.01± 0.06	1.00± 0.07	17.62± 0.23	24.73± 0.38
microsatellite/ LINE-1	1	1.00± 0.07	1.09± 0.06	1.26± 0.16	1.00± 0.12	0.80± 0.09	0.01± 0.01	1.00± 0.11	0.15± 0.05	0.24± 0.06	1.00± 0.06	1.47± 0.09	1.39± 0.15
	2	1.00±	1.64±	3.87±	1.00±	1.32±	1.10±	1.00±	1.21±	0.04±	1.00±	5.22±	5.05±

		0.07	0.13	0.14	0.10	0.10	0.14	0.10	0.05	0.02	0.09	0.19	0.20
Sod1	1	1.00± 0.08	1.01± 0.13	1.72± 0.11	1.00± 0.08	1.03± 0.06	0.94± 0.10	1.00± 0.08	1.19± 0.13	0.39± 0.09	1.00± 0.05	2.34± 0.18	3.52± 0.17
	2	1.00± 0.10	1.46± 0.12	2.62± 0.13	1.00± 0.07	1.73± 0.06	0.67± 0.13	1.00± 0.09	0.29± 0.05	0.27± 0.10	1.00± 0.10	5.48± 0.15	7.30± 0.15
Prx5	1	1.00± 0.12	0.01± 0.02	0.41± 0.04	1.00± 0.06	0.01± 0.01	0.23± 0.06	1.00± 0.08	0.22± 0.07	0.21± 0.05	1.00± 0.05	0.13± 0.08	0.23± 0.04
	2	1.00± 0.12	0.28± 0.05	0.99± 0.15	1.00± 0.05	0.16± 0.07	0.15± 0.06	1.00± 0.08	0.68± 0.07	0.08± 0.05	1.00± 0.08	0.37± 0.10	0.56± 0.11
Gadd45	1	1.00± 0.09	1.04± 0.08	1.02± 0.12	1.00± 0.15	0.85± 0.08	1.62± 0.12	1.00± 0.06	0.29± 0.07	1.14± 0.10	1.00± 0.09	0.93± 0.11	0.25± 0.05
	2	1.00± 0.08	1.34± 0.11	0.42± 0.10	1.00± 0.05	1.16± 0.09	1.21± 0.12	1.00± 0.05	0.97± 0.14	1.44± 0.10	1.00± 0.09	1.36± 0.10	0.72± 0.06
Xpc	1	1.00± 0.09	0.57± 0.07	2.15± 0.12	1.00± 0.06	2.18± 0.17	1.55± 0.13	1.00± 0.05	0.46± 0.07	0.34± 0.07	1.00± 0.13	2.00± 0.15	3.27± 0.15
	2	1.00± 0.09	1.32± 0.07	3.41± 0.18	1.00± 0.07	4.26± 0.18	2.48± 0.18	1.00± 0.11	0.87± 0.10	0.01± 0.03	1.00± 0.10	3.20± 0.11	7.75± 0.12
Ku80	1	1.00± 0.07	1.25± 0.13	3.02± 0.18	1.00± 0.08	4.15± 0.20	7.55± 0.23	1.00± 0.10	0.13± 0.04	0.43± 0.11	1.00± 0.05	5.15± 0.14	3.18± 0.17
	2	1.00± 0.14	1.51± 0.13	4.63± 0.14	1.00± 0.08	2.23± 0.10	12.55± 0.23	1.00± 0.09	0.87± 0.13	0.83± 0.11	1.00± 0.06	6.53± 0.17	7.70± 0.20
spn-B	1	1.00± 0.01	5.99± 0.11	5.64± 0.24	1.00± 0.09	2.51± 0.18	15.69± 0.26	1.00± 0.09	0.95± 0.06	0.85± 0.06	1.00± 0.10	17.31± 0.27	18.96± 0.31
	2	1.00± 0.08	3.24± 0.17	4.12± 0.19	1.00± 0.10	1.55± 0.09	6.95± 0.19	1.00± 0.12	1.32± 0.15	1.50± 0.06	1.00± 0.08	11.68± 0.21	11.60± 0.25
Hsp27	1	1.00± 0.10	1.35± 0.10	1.25± 0.18	1.00± 0.09	1.85± 0.07	2.67± 0.18	1.00± 0.06	1.89± 0.12	0.56± 0.08	1.00± 0.12	2.45± 0.18	4.58± 0.16
	2	1.00± 0.09	1.80± 0.10	2.03± 0.17	1.00± 0.07	3.18± 0.16	4.96± 0.18	1.00± 0.06	1.52± 0.14	0.90± 0.08	1.00± 0.08	3.36± 0.18	3.05± 0.19
Hsp68	1	1.00± 0.07	1.08± 0.15	2.93± 0.18	1.00± 0.05	12.69± 0.27	10.53± 0.26	1.00± 0.05	0.38± 0.03	0.31± 0.05	1.00± 0.09	6.44± 0.27	7.87± 0.20
	2	1.00± 0.06	0.64± 0.08	4.57± 0.28	1.00± 0.12	6.97± 0.12	16.35± 0.24	1.00± 0.14	1.30± 0.13	0.61± 0.05	1.00± 0.07	4.20± 0.15	5.98± 0.27
Atg1	1	1.00± 0.06	1.85± 0.14	2.74± 0.14	1.00± 0.10	2.10± 0.12	1.12± 0.13	1.00± 0.08	0.57± 0.10	0.55± 0.13	1.00± 0.07	7.56± 0.20	6.31± 0.21
	2	1.00± 0.04	1.21± 0.15	1.98± 0.14	1.00± 0.09	1.62± 0.15	1.67± 0.11	1.00± 0.07	0.89± 0.16	0.19± 0.04	1.00± 0.09	5.47± 0.16	9.55± 0.21
Atg5	1	1.00± 0.09	0.74± 0.09	0.57± 0.05	1.00± 0.07	0.59± 0.09	2.22± 0.20	1.00± 0.08	1.25± 0.07	0.95± 0.12	1.00± 0.04	0.56± 0.07	0.36± 0.05
	2	1.00± 0.08	1.19± 0.10	0.92± 0.08	1.00± 0.06	1.41± 0.09	4.12± 0.20	1.00± 0.09	2.22± 0.16	1.71± 0.09	1.00± 0.11	1.11± 0.06	1.32± 0.12
Ire1	1	1.00± 0.09	0.82± 0.11	1.75± 0.14	1.00± 0.06	1.05± 0.14	2.61± 0.11	1.00± 0.09	0.43± 0.06	0.32± 0.08	1.00± 0.07	2.32± 0.11	2.71± 0.17
	2	1.00± 0.10	1.27± 0.13	3.24± 0.24	1.00± 0.08	2.25± 0.13	4.40± 0.26	1.00± 0.08	0.67± 0.08	0.68± 0.11	1.00± 0.10	4.74± 0.10	6.71± 0.27

Table S5. Mean relative gene expression of flies with tissue-specific *AGO1* knockdown in the condition of γ-irradiation.

**Genotype**	***GS-elav>RNAi-AGO1***								***GS-S106>RNAi-AGO1***							
**Sex**	**Males**				**Females**				**Males**				**Females**			
**RU486**	**-**	**+**	**-**	**+**	**-**	**+**	**-**	**+**	**-**	**+**	**-**	**+**	**-**	**+**	**-**	**+**
**IR**	**0**	**0**	**700**	**700**	**0**	**0**	**700**	**700**	**0**	**0**	**700**	**700**	**0**	**0**	**700**	**700**
HeT-A1	1.00±0.32	0.52±0.08	0.85±0.30	0.42±0.08	1.00±0.18	0.96±0.19	2.14±0.17	1.59±0.09	1.00±0.38	0.31±0.06	1.20±0.04	0.26±0.06	1.00±0.35	0.11±0.04	1.29±0.29	0.21±0.05
R1-element	1.00±0.28	0.39±0.37	0.48±0.17	0.17±0.08	1.00±0.22	0.28±0.23	0.43±0.11	0.24±0.13	1.00±0.29	0.19±0.07	0.99±0.22	0.08±0.05	1.00±0.37	0.05±0.07	0.69±0.21	0.11±0.04
Rt1a	1.00±0.27	0.25±0.18	0.81±0.33	0.30±0.28	1.00±0.15	0.64±0.17	4.75±0.15	0.58±0.08	1.00±0.37	0.25±0.08	0.92±0.09	0.08±0.09	1.00±0.36	0.06±0.06	0.78±0.49	0.16±0.11
1731	1.00±0.18	0.44±0.06	0.93±0.20	0.90±0.28	1.00±0.34	0.52±0.36	5.60±0.18	1.86±0.20	1.00±0.08	0.12±0.09	0.56±0.08	0.43±0.25	1.00±0.26	0.16±0.12	0.94±0.32	0.35±0.04
412	1.00±0.18	0.49±0.10	0.96±0.36	0.69±0.16	1.00±0.07	0.60±0.38	2.75±0.22	1.30±0.15	1.00±0.29	0.11±0.07	1.00±0.09	0.26±0.08	1.00±0.10	0.53±0.08	1.51±0.07	0.97±0.28
blood	1.00±0.16	0.60±0.22	0.82±0.23	0.69±0.15	1.00±0.18	0.81±0.15	1.57±0.28	1.53±0.34	1.00±0.14	0.30±0.11	1.29±0.16	0.58±0.20	1.00±0.22	0.17±0.09	0.89±0.15	0.29±0.05
opus	1.00±0.25	0.50±0.29	2.37±0.61	1.24±0.40	1.00±0.17	1.28±0.36	2.58±0.27	1.10±0.26	1.00±0.08	0.15±0.06	0.58±0.26	0.07±0.04	1.00±0.50	0.07±0.05	0.69±0.55	0.15±0.07
roo	1.00±0.26	0.40±0.23	0.52±0.37	0.30±0.17	1.00±0.05	0.75±0.36	2.53±0.07	1.06±0.07	1.00±0.10	0.29±0.08	1.41±0.15	0.26±0.12	1.00±0.39	0.12±0.09	0.99±0.15	0.27±0.02
microsatellite/ LINE-1	1.00±0.27	0.64±0.16	0.77±0.30	0.53±0.25	1.00±0.24	0.78±0.05	2.03±0.18	1.25±0.27	1.00±0.29	0.47±0.20	1.60±0.11	0.62±0.21	1.00±0.48	0.33±0.19	1.14±0.48	0.45±0.28
Sod1	1.00±0.12	0.99±0.17	1.02±0.17	1.22±0.22	1.00±0.14	0.90±0.16	0.79±0.29	1.03±0.15	1.00±0.22	2.20±0.27	0.93±0.15	1.63±0.26	1.00±0.17	2.26±0.29	1.82±0.28	1.85±0.18
Prx5	1.00±0.13	0.93±0.17	2.79±0.15	1.38±0.17	1.00±0.17	2.19±0.19	1.00±0.19	0.75±0.24	1.00±0.19	11.07±0.18	0.72±0.17	6.32±0.18	1.00±0.26	3.01±0.17	4.58±0.29	1.24±0.29
Gadd45	1.00±0.24	0.73±0.17	6.79±0.22	6.86±0.27	1.00±0.20	2.26±0.17	6.40±0.18	8.98±0.13	1.00±0.15	4.22±0.15	2.73±0.28	2.82±0.17	1.00±0.14	0.73±0.24	1.35±0.14	0.62±0.14
Xpc	1.00±0.32	1.15±0.44	6.59±0.10	6.78±0.71	1.00±0.25	0.81±0.17	5.64±0.14	5.52±0.25	1.00±0.15	0.61±0.25	4.12±0.36	7.47±0.26	1.00±0.16	0.81±0.16	2.02±0.25	1.68±0.17
Ku80	1.00±0.24	1.03±0.16	2.30±0.15	2.70±0.34	1.00±0.18	1.67±0.31	1.10±0.27	0.73±0.19	1.00±0.19	14.28±0.17	3.64±0.09	3.55±0.20	1.00±0.15	2.39±0.18	2.34±0.32	2.58±0.49
spn-B	1.00±0.12	0.95±0.16	6.68±0.16	6.54±0.18	1.00±0.20	1.08±0.16	14.27±0.47	15.91±0.30	1.00±0.15	1.41±0.15	15.29±0.15	13.51±0.11	1.00±0.18	0.80±0.20	3.91±0.29	3.55±0.24
Hsp27	1.00±0.17	0.64±0.21	4.73±0.29	2.41±0.18	1.00±0.19	4.34±0.38	0.79±0.13	0.51±0.15	1.00±0.18	6.29±0.24	1.78±0.14	1.41±0.10	1.00±0.17	1.19±0.12	1.70±0.14	1.66±0.27
Hsp68	1.00±0.20	1.12±0.15	8.11±0.18	14.02±0.24	1.00±0.13	3.22±0.19	9.58±0.37	15.61±0.06	1.00±0.14	11.87±0.17	6.09±0.19	3.64±0.26	1.00±0.10	0.94±0.17	0.84±0.27	5.61±0.25
Atg1	1.00±0.24	0.80±0.21	1.68±0.19	1.74±0.16	1.00±0.12	1.05±0.31	1.49±0.28	1.40±0.19	1.00±0.15	1.69±0.19	2.21±0.16	1.15±0.20	1.00±0.12	0.87±0.19	0.92±0.19	0.73±0.18
Atg5	1.00±0.17	0.74±0.16	0.90±0.16	0.95±0.18	1.00±0.17	1.08±0.09	0.79±0.08	1.02±0.18	1.00±0.19	1.15±0.20	0.34±0.09	0.92±0.19	1.00±0.15	0.58±0.16	1.47±0.18	0.96±0.13
Ire1	1.00±0.15	0.87±0.15	4.78±0.40	4.97±0.17	1.00±0.18	0.79±0.27	0.67±0.38	0.98±0.16	1.00±0.14	5.04±0.25	1.67±0.14	2.14±0.19	1.00±0.19	0.83±0.21	2.16±0.20	1.02±0.13

RU486 – mifepristone treatment

IR – ionizing irradiation dose (Gy)

Table S6. Mean relative gene expression of flies with tissue-specific *AGO2* knockdown in the condition of γ-irradiation.

**Genotype**	***GS-elav>RNAi-AGO2***								***GS-S106>RNAi-AGO2***							
**Sex**	**Males**				**Females**				**Males**				**Females**			
**RU486**	**-**	**+**	**-**	**+**	**-**	**+**	**-**	**+**	**-**	**+**	**-**	**+**	**-**	**+**	**-**	**+**
**IR**	**0**	**0**	**700**	**700**	**0**	**0**	**700**	**700**	**0**	**0**	**700**	**700**	**0**	**0**	**700**	**700**
HeT-A1	1.00±0.16	0.97±0.22	1.03±0.23	0.60±0.15	1.00±0.24	0.48±0.11	0.46±0.16	1.94±0.20	1.00±0.18	0.48±0.15	0.63±0.28	1.38±0.34	1.00±0.22	0.59±0.09	0.48±0.15	0.69±0.07
R1-element	1.00±0.20	0.80±0.08	0.40±0.27	0.34±0.18	1.00±0.19	0.35±0.24	0.26±0.19	1.44±0.18	1.00±0.14	0.21±0.06	0.31±0.20	0.98±0.19	1.00±0.13	0.42±0.09	0.50±0.18	0.52±0.10
Rt1a	1.00±0.23	0.69±0.13	0.76±0.12	0.31±0.12	1.00±0.09	0.36±0.11	0.20±0.29	1.26±0.23	1.00±0.10	0.17±0.13	0.51±0.26	0.75±0.22	1.00±0.15	0.74±0.17	0.51±0.09	0.70±0.27
1731	1.00±0.21	0.37±0.26	0.94±0.08	0.79±0.10	1.00±0.26	0.17±0.31	0.22±0.30	0.43±0.25	1.00±0.16	0.81±0.39	1.90±0.53	3.04±0.45	1.00±0.24	0.55±0.14	0.47±0.19	0.29±0.06
412	1.00±0.06	0.81±0.16	1.11±0.26	0.50±0.19	1.00±0.25	1.02±0.17	0.26±0.18	1.31±0.28	1.00±0.16	0.37±0.14	0.68±0.19	1.58±0.27	1.00±0.05	0.75±0.05	0.89±0.15	0.51±0.15
blood	1.00±0.14	0.57±0.25	1.58±0.18	0.96±0.14	1.00±0.18	0.15±0.17	0.66±0.18	0.33±0.20	1.00±0.17	0.45±0.15	0.83±0.10	1.38±0.25	1.00±0.24	0.51±0.07	0.75±0.18	0.56±0.17
opus	1.00±0.13	0.55±0.16	1.89±0.28	0.90±0.14	1.00±0.18	0.38±0.16	0.34±0.15	0.87±0.15	1.00±0.18	0.20±0.14	0.24±0.40	0.76±0.20	1.00±0.16	0.33±0.16	0.68±0.15	0.15±0.09
roo	1.00±0.17	0.66±0.25	1.04±0.08	0.44±0.08	1.00±0.23	0.19±0.15	0.36±0.16	0.50±0.19	1.00±0.10	0.44±0.15	0.66±0.15	1.01±0.38	1.00±0.11	0.39±0.16	0.59±0.19	0.63±0.18
microsatellite/ LINE-1	1.00±0.12	0.48±0.13	1.30±0.07	0.73±0.25	1.00±0.15	0.65±0.15	0.54±0.34	1.23±0.38	1.00±0.17	0.68±0.17	0.70±0.20	1.06±0.26	1.00±0.07	0.49±0.05	0.86±0.24	0.22±0.07
Sod1	1.00±0.25	1.49±0.25	1.48±0.17	0.96±0.19	1.00±0.16	0.86±0.19	0.59±0.25	0.63±0.15	1.00±0.10	2.46±0.12	1.93±0.20	1.89±0.18	1.00±0.19	0.76±0.17	0.45±0.18	0.49±0.25
Prx5	1.00±0.24	1.32±0.28	1.21±0.19	0.64±0.20	1.00±0.18	0.96±0.20	0.46±0.28	0.53±0.18	1.00±0.15	1.56±0.16	6.75±0.25	5.04±0.24	1.00±0.15	0.38±0.16	1.00±0.38	1.09±0.26
Gadd45	1.00±0.17	0.50±0.18	17.25±0.36	10.21 ±0.26	1.00±0.18	0.65±0.19	3.25±0.16	3.20±0.18	1.00±0.19	0.63±0.27	2.50±0.19	1.82±0.16	1.00±0.16	0.18±0.13	1.01±0.26	0.32±0.15
Xpc	1.00±0.16	1.69±0.19	8.29±0.24	5.27±0.21	1.00±0.17	1.16±0.22	4.58±0.19	9.29±0.17	1.00±0.16	0.83±0.10	4.65±0.37	4.52±0.14	1.00±0.26	0.44±0.18	1.37±0.34	0.90±0.18
Ku80	1.00±0.18	1.21±0.23	2.57±0.18	1.53±0.25	1.00±0.19	1.57±0.27	0.80±0.26	1.38±0.18	1.00±0.16	1.70±0.15	0.86±0.17	0.55±0.27	1.00±0.16	0.69±0.23	0.59±0.19	0.37±0.18
spn-B	1.00±0.24	0.67±0.17	17.45±0.24	11.24 ±0.24	1.00±0.22	0.97±0.24	16.48±0.15	13.14 ±0.24	1.00±0.15	0.41±0.27	8.96±0.19	6.78±0.25	1.00±0.14	0.43±0.12	5.12±0.13	2.91±0.41
Hsp27	1.00±0.19	0.61±0.28	1.41±0.14	0.96±0.20	1.00±0.13	0.85±0.26	0.55±0.19	1.17±0.20	1.00±0.27	1.19±0.15	0.62±0.28	1.00±0.16	1.00±0.25	0.29±0.15	0.42±0.18	0.30±0.46
Hsp68	1.00±0.11	0.68±0.28	9.83±0.43	9.94±0.29	1.00±0.12	1.50±0.16	7.23±0.18	8.17±0.29	1.00±0.14	0.47±0.15	0.68±0.30	1.70±0.16	1.00±0.22	0.86±0.28	0.82±0.11	0.57±0.11
Atg1	1.00±0.25	1.06±0.13	1.31±0.19	1.35±0.17	1.00±0.19	0.70±0.17	1.22±0.18	1.36±0.25	1.00±0.14	0.55±0.19	0.95±0.28	1.13±0.18	1.00±0.20	0.37±0.16	1.31±0.26	0.74±0.24
Atg5	1.00±0.20	0.92±0.26	0.94±0.16	0.62±0.25	1.00±0.18	1.17±0.14	1.19±0.16	1.48±0.11	1.00±0.28	1.91±0.19	1.49±0.26	0.79±0.16	1.00±0.18	0.48±0.33	0.58±0.15	0.24±0.16
Ire1	1.00±0.13	0.73±0.20	1.92±0.13	1.54±0.15	1.00±0.19	0.82±0.20	0.97±0.24	1.44±0.19	1.00±0.25	1.31±0.15	1.97±0.19	1.48±0.24	1.00±0.25	0.55±0.20	1.48±0.18	0.53±0.19

RU486 – mifepristone treatment
IR – ionizing irradiation dose (Gy)

Table S7. Mean relative gene expression of flies with tissue-specific *AGO3* knockdown in the condition of γ-irradiation.

Genotype	*GS-elav>RNAi-AGO3*								*GS-S106>RNAi-AGO3*							
**Sex**	**Males**				**Females**				**Males**				**Females**			
**RU486**	-	+	-	+	-	+	-	+	-	+	-	+	-	+	-	+
**IR**	0	0	700	700	0	0	700	700	0	0	700	700	0	0	700	700
HeT-A1	1.00±0.17	0.84±0.16	0.79±0.19	0.43±0.14	1.00±0.15	1.57±0.14	1.38±0.14	1.35±0.20	1.00±0.18	1.26±0.17	1.51±0.23	1.63±0.44	1.00±0.16	2.35±0.30	3.73±0.30	0.96±0.14
R1-element	1.00±0.21	1.01±0.19	0.35±0.08	0.21±0.16	1.00±0.20	3.61±0.27	2.76±0.14	1.31±0.29	1.00±0.26	1.09±0.14	1.48±0.17	1.56±0.13	1.00±0.20	3.56±0.23	6.25±0.22	0.77±0.26
Rt1a	1.00±0.19	1.25±0.18	0.48±0.16	0.35±0.16	1.00±0.15	2.60±0.15	1.88±0.15	1.23±0.21	1.00±0.23	1.07±0.17	1.41±0.25	1.38±0.16	1.00±0.12	2.95±0.32	5.60±0.25	0.71±0.17
1731	1.00±0.15	1.17±0.13	0.90±0.26	0.79±0.19	1.00±0.16	1.64±0.18	1.46±0.19	1.28±0.16	1.00±0.14	1.51±0.20	1.23±0.18	1.60±0.16	1.00±0.25	2.13±0.25	2.26±0.24	0.73±0.19
412	1.00±0.25	1.22±0.29	1.16±0.15	1.09±0.13	1.00±0.18	2.26±0.20	1.37±0.23	1.50±0.15	1.00±0.26	1.30±0.16	0.77±0.19	0.76±0.16	1.00±0.14	2.24±0.26	2.32±0.15	0.95±0.20
blood	1.00±0.26	1.77±0.17	2.65±0.39	1.53±0.26	1.00±0.24	1.04±0.13	0.92±0.16	1.17±0.17	1.00±0.12	1.26±0.37	1.50±0.15	1.57±0.20	1.00±0.30	1.97±0.19	2.07±0.12	1.28±0.21
opus	1.00±0.13	1.34±0.10	1.49±0.27	0.97±0.13	1.00±0.17	2.01±0.30	1.27±0.11	1.71±0.16	1.00±0.16	1.13±0.18	1.30±0.21	1.43±0.14	1.00±0.30	2.87±0.15	5.91±0.17	0.94±0.21
roo	1.00±0.26	0.95±0.15	0.93±0.14	0.63±0.20	1.00±0.27	1.17±0.36	1.42±0.25	0.92±0.29	1.00±0.35	1.55±0.16	1.20±0.19	1.13±0.25	1.00±0.15	1.72±0.10	2.50±0.16	1.05±0.25
microsatellite/ LINE-1	1.00±0.16	1.17±0.18	1.15±0.16	0.96±0.15	1.00±0.19	1.37±0.16	1.26±0.15	1.24±0.13	1.00±0.23	1.82±0.24	1.24±0.27	1.22±0.20	1.00±0.16	1.89±0.28	2.87±0.22	1.74±0.19
Sod1	1.00±0.20	1.02±0.35	1.28±0.25	1.17±0.18	1.00±0.19	0.90±0.18	0.81±0.16	1.63±0.16	1.00±0.26	0.95±0.28	1.08±0.13	1.19±0.25	1.00±0.25	1.07±0.20	3.55±0.20	1.16±0.23
Prx5	1.00±0.17	1.42±0.25	1.42±0.18	3.80±0.12	1.00±0.25	5.03±0.23	1.07±0.21	5.65±0.38	1.00±0.28	2.77±0.17	1.85±0.16	7.78±0.25	1.00±0.19	8.52±0.21	0.94±0.19	18.72±0.49
Gadd45	1.00±0.18	1.22±0.19	18.85±0.39	16.62±0.39	1.00±0.16	1.35±0.19	3.87±0.18	11.19±0.31	1.00±0.15	0.75±0.13	3.80±0.27	3.09±0.24	1.00±0.16	1.40±0.11	4.04±0.24	4.34±0.23
Xpc	1.00±0.15	0.90±0.16	7.47±0.26	6.89±0.27	1.00±0.15	1.01±0.16	6.63±0.15	9.46±0.20	1.00±0.28	0.72±0.24	5.72±0.25	5.23±0.25	1.00±0.12	1.15±0.21	1.69±0.24	4.31±0.35
Ku80	1.00±0.17	1.13±0.17	3.45±0.20	5.25±0.31	1.00±0.16	1.06±0.16	1.52±0.18	3.30±0.19	1.00±0.15	1.34±0.14	1.66±0.17	1.27±0.27	1.00±0.17	1.52±0.24	1.52±0.15	1.53±0.16
spn-B	1.00±0.19	0.80±0.14	28.72±0.33	21.04±0.37	1.00±0.19	0.75±0.15	25.98±0.33	32.18±0.44	1.00±0.18	0.51±0.13	11.76±0.23	7.53±0.15	1.00±0.22	1.52±0.21	18.67±0.38	19.99±0.40
Hsp27	1.00±0.17	1.13±0.14	1.80±0.13	2.72±0.38	1.00±0.34	1.94±0.28	1.85±0.23	2.46±0.17	1.00±0.20	0.72±0.10	1.48±0.27	1.38±0.26	1.00±0.26	1.14±0.19	3.66±0.22	1.80±0.19
Hsp68	1.00±0.28	1.25±0.19	5.12±0.18	9.93±0.25	1.00±0.23	1.40±0.32	5.34±0.15	6.81±0.24	1.00±0.13	0.30±0.17	5.53±0.18	3.75±0.17	1.00±0.16	0.73±0.13	16.07±0.36	3.28±0.25
Atg1	1.00±0.15	1.36±0.16	1.60±0.31	0.79±0.17	1.00±0.26	0.95±0.26	1.44±0.16	1.94±0.15	1.00±0.25	0.62±0.15	0.83±0.26	0.77±0.26	1.00±0.23	1.58±0.20	6.85±0.18	3.58±0.25
Atg5	1.00±0.15	0.99±0.20	0.97±0.16	0.85±0.19	1.00±0.20	1.19±0.18	0.86±0.20	1.87±0.17	1.00±0.26	0.77±0.19	0.87±0.33	0.88±0.28	1.00±0.16	2.04±0.20	1.18±0.20	3.25±0.25
Ire1	1.00±0.15	1.79±0.12	5.58±0.15	4.94±0.26	1.00±0.21	0.56±0.12	0.76±0.24	2.70±0.28	1.00±0.19	1.45±0.14	2.42±0.15	1.32±0.38	1.00±0.18	0.83±0.27	2.73±0.30	3.03±0.27

RU486 – mifepristone treatment
IR – ionizing irradiation dose (Gy)

Table S8. Mean relative gene expression of flies with tissue-specific *piwi* knockdown in the condition of γ-irradiation.

**Genotype**	*GS-elav>RNAi-piwi*								*GS-S106>RNAi-piwi*							
**Sex**	**Males**				**Females**				**Males**				**Females**			
**RU486**	**-**	**+**	**-**	**+**	**-**	**+**	**-**	**+**	**-**	**+**	**-**	**+**	**-**	**+**	**-**	**+**
**IR**	**0**	**0**	**700**	**700**	**0**	**0**	**700**	**700**	**0**	**0**	**700**	**700**	**0**	**0**	**700**	**700**
HeT-A1	1.00± 0.10	2.51± 0.14	2.03± 0.13	0.71± 0.12	1.00± 0.18	1.25± 0.17	1.58± 0.12	0.92± 0.11	1.00± 0.09	1.85± 0.13	1.53± 0.23	1.43± 0.10	1.00± 0.15	3.49± 0.18	6.67± 0.37	1.37± 0.10
R1-element	1.00± 0.18	4.42± 0.25	1.97± 0.25	0.66± 0.14	1.00± 0.07	1.35± 0.16	1.91± 0.25	0.27± 0.20	1.00± 0.15	2.35± 0.12	2.19± 0.26	0.99± 0.16	1.00± 0.14	6.12± 0.14	8.07± 0.28	1.45± 0.08
Rt1a	1.00± 0.06	2.93± 0.16	2.06± 0.15	0.54± 0.08	1.00± 0.21	1.93± 0.13	1.66± 0.16	0.38± 0.19	1.00± 0.18	2.55± 0.15	2.94± 0.10	1.54± 0.16	1.00± 0.20	6.58± 0.41	9.34± 0.29	1.87± 0.40
1731	1.00± 0.13	1.55± 0.16	1.21± 0.07	0.83± 0.18	1.00± 0.23	1.20± 0.27	1.12± 0.24	0.34± 0.21	1.00± 0.17	0.97± 0.17	1.79± 0.22	1.31± 0.28	1.00± 0.23	3.00± 0.29	5.74± 0.16	1.09± 0.12
412	1.00± 0.05	2.01± 0.09	1.82± 0.22	0.84± 0.08	1.00± 0.06	1.68± 0.08	1.81± 0.18	1.12± 0.15	1.00± 0.05	2.04± 0.04	2.48± 0.06	2.12± 0.04	1.00± 0.28	3.83± 0.17	3.55± 0.12	1.62± 0.09
blood	1.00± 0.19	2.11± 0.31	2.32± 0.18	1.30± 0.28	1.00± 0.22	1.03± 0.29	1.20± 0.36	1.16± 0.25	1.00± 0.19	1.64± 0.15	2.06± 0.21	1.39± 0.14	1.00± 0.10	2.76± 0.23	2.84± 0.22	1.48± 0.48
opus	1.00± 0.08	1.18± 0.26	1.48± 0.12	0.81± 0.06	1.00± 0.05	2.44± 0.10	1.91± 0.10	1.05± 0.18	1.00± 0.06	2.42± 0.07	2.09± 0.10	1.53± 0.06	1.00± 0.13	7.15± 0.09	6.97± 0.34	2.11± 0.36
roo	1.00± 0.19	2.56± 0.13	2.08± 0.27	0.87± 0.15	1.00± 0.12	0.97± 0.22	1.43± 0.22	0.63± 0.20	1.00± 0.14	2.11± 0.16	2.56± 0.24	1.39± 0.13	1.00± 0.31	3.63± 0.21	5.87± 0.25	1.53± 0.26
microsatellite/ LINE-1	1.00± 0.14	2.09± 0.19	1.72± 0.22	0.93± 0.06	1.00± 0.15	0.88± 0.09	1.33± 0.27	0.80± 0.29	1.00± 0.15	1.20± 0.12	1.16± 0.15	0.78± 0.13	1.00± 0.48	2.67± 0.24	2.04± 0.14	1.58± 0.33
Sod1	1.00± 0.17	0.89± 0.18	0.85± 0.16	0.74± 0.19	1.00± 0.19	1.57± 0.20	0.81± 0.26	1.42± 0.25	1.00± 0.18	1.09± 0.18	1.11± 0.18	1.56± 0.18	1.00± 0.15	1.23± 0.25	1.17± 0.18	1.62± 0.26
Prx5	1.00± 0.20	1.25± 0.19	0.96± 0.25	1.00± 0.29	1.00± 0.17	4.38± 0.15	1.30± 0.24	2.40± 0.16	1.00± 0.26	1.67± 0.21	1.23± 0.26	2.30± 0.15	1.00± 0.25	1.43± 0.17	1.23± 0.18	1.22± 0.26
Gadd45	1.00± 0.16	1.81± 0.23	12.24 ±0.29	13.21± 0.28	1.00± 0.26	2.72± 0.33	5.04± 0.22	7.11± 0.15	1.00± 0.13	1.36± 0.20	2.68± 0.27	2.80± 0.21	1.00± 0.14	7.07± 0.41	9.18± 0.37	9.15± 0.39
Xpc	1.00± 0.17	0.77± 0.10	5.79± 0.13	4.71± 0.22	1.00± 0.27	1.28± 0.31	6.18± 0.19	5.96± 0.24	1.00± 0.15	0.88± 0.19	4.55± 0.20	4.75± 0.19	1.00± 0.14	1.96± 0.20	3.06± 0.13	2.65± 0.25
Ku80	1.00± 0.19	3.20± 0.17	3.43± 0.29	2.91± 0.16	1.00± 0.21	1.42± 0.17	1.28± 0.23	1.47± 0.13	1.00± 0.11	3.09± 0.31	1.60± 0.15	1.18± 0.22	1.00± 0.22	0.77± 0.21	1.03± 0.15	1.12± 0.21
spn-B	1.00± 0.19	1.00± 0.17	21.18 ±0.37	20.30± 0.34	1.00± 0.26	1.52± 0.16	16.77 ±0.42	22.19± 0.27	1.00± 0.19	0.98± 0.15	16.23 ±0.35	10.81± 0.30	1.00± 0.22	3.07± 0.16	22.49 ±0.38	13.18 ±0.38
Hsp27	1.00± 0.18	0.77± 0.15	1.41± 0.27	2.04± 0.17	1.00± 0.26	1.69± 0.17	1.08± 0.27	1.06± 0.26	1.00± 0.16	1.37± 0.20	1.33± 0.28	1.48± 0.17	1.00± 0.17	1.43± 0.14	1.42± 0.28	1.03± 0.22
Hsp68	1.00± 0.20	1.85± 0.21	7.04± 0.32	10.00± 0.18	1.00± 0.30	0.71± 0.18	1.74± 0.31	5.76± 0.37	1.00± 0.18	1.14± 0.21	2.32± 0.19	2.69± 0.19	1.00± 0.18	1.51± 0.17	5.80± 0.27	2.38± 0.25
Atg1	1.00± 0.14	2.22± 0.18	1.58± 0.24	2.18± 0.27	1.00± 0.17	1.34± 0.15	1.80± 0.31	1.71± 0.25	1.00± 0.25	0.68± 0.18	1.00± 0.18	0.87± 0.23	1.00± 0.19	3.71± 0.23	4.49± 0.33	2.63± 0.29
Atg5	1.00± 0.24	1.28± 0.25	0.98± 0.13	0.96± 0.21	1.00± 0.26	1.50± 0.20	1.38± 0.19	1.61± 0.15	1.00± 0.16	0.84± 0.23	0.72± 0.24	0.73± 0.13	1.00± 0.26	0.67± 0.20	0.77± 0.20	0.87± 0.22
Ire1	1.00± 0.20	4.06± 0.15	5.39± 0.25	6.35± 0.18	1.00± 0.26	2.29± 0.16	1.31± 0.17	2.97± 0.28	1.00± 0.15	0.89± 0.20	1.67± 0.16	1.58± 0.24	1.00± 0.14	1.27± 0.24	2.16± 0.17	1.48± 0.34

RU486 – mifepristone treatment
IR – ionizing irradiation dose (Gy)

Table S9. *Drosophila melanogaster* strains.

Strain	Genotype	Description	Source
Canton-S	Wild-type strain	Wild-type strain	Bloomington *Drosophila* Stock Center, Bloomington, IN, USA (#64349)
RNAi-AGO1	*y[1] v[1]; P{y[+t7.7] v[+t1.8]=TRiP.HM04006}attP2*	Expresses dsRNA for RNAi of *AGO1* under *UAS* control in the *VALIUM1* vector	Bloomington *Drosophila* Stock Center, Bloomington, IN, USA (#31700)
RNAi-AGO2	*y[1] sc[*] v[1] sev[21]; P{y[+t7.7] v[+t1.8]=TRiP.HMS00108}attP2*	Expresses dsRNA for RNAi of *AGO2* under *UAS* control in the *VALIUM20* vector	Bloomington *Drosophila* Stock Center, Bloomington, IN, USA (#34799)
RNAi-AGO3	*y[1] v[1]; P{y[+t7.7] v[+t1.8]=TRiP.HMC02938}attP40*	Expresses dsRNA for RNAi of *AGO3* under *UAS* control in the *VALIUM20* vector	Bloomington *Drosophila* Stock Center, Bloomington, IN, USA (#44543)
RNAi-piwi	*y[1] v[1]; P{y[+t7.7] v[+t1.8]=TRiP.HMJ21827}attP40/ CyO*	Expresses dsRNA for RNAi of *piwi* under *UAS* control in the *VALIUM20* vector	Bloomington *Drosophila* Stock Center, Bloomington, IN, USA (#57819)
GS-elav	*y[1] w[*]; P{w[+mC]=elav-Switch.O}GSG301*	Expresses steroid-activated GAL4 in the nervous system	Bloomington *Drosophila* Stock Center, Bloomington, IN, USA (#43642)
GS-S106	*w[1118]; P{w[+mW.hs]=Switch1}106*	Expresses GAL4 fused to steroid receptor ligand binding domain in the adult fat body	Bloomington *Drosophila* Stock Center, Bloomington, IN, USA (#8151)
GS-TIGS-2	*P{Switch-unk}TIGS-2*	Expresses GAL4 in the digestive system	Courtesy of Dr. Laurent Seroude (Queen's University, Kingston, ON, Canada) from Dr. Scott Pletcher (University of Michigan, Ann Arbor, MI, USA)
GS-Mhc	*w; Sp/CyO; P{MHC-GeneSwitch}*	Expresses GAL4 in muscles	

All strains are maintained in the Collection of Laboratory Strains of Fruit Flies *Drosophila* (IB FRC Komi SC UB RAS, Syktyvkar, Russia).

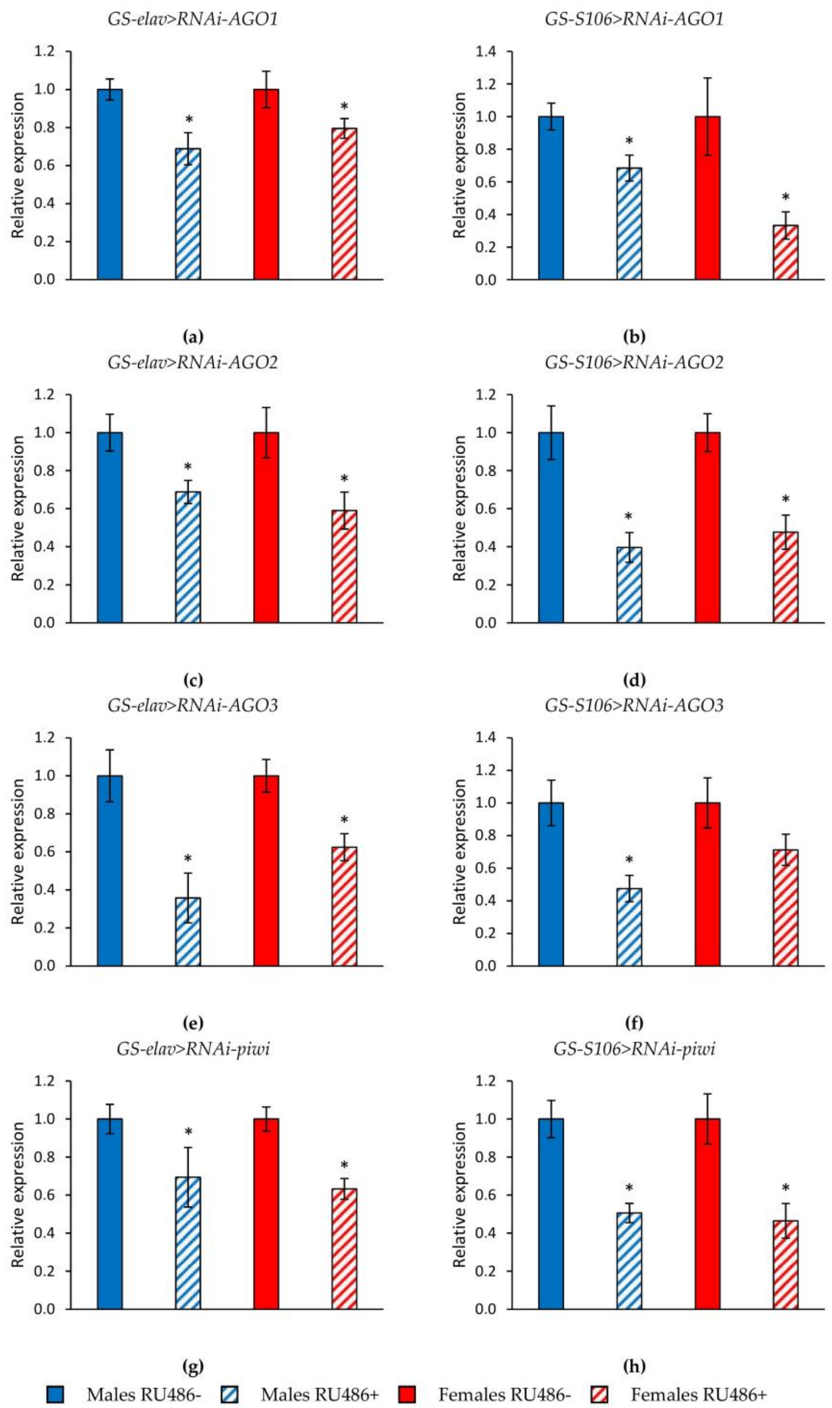

Figure S4. Knockdown of *AGO1* (**a, b**), *AGO2* (**c, d**), *AGO3* (**e, f**), and *piwi* (**g, h**) in investigated flies. Differences between relative expression levels of flies with *Argonaute* genes' knockdown (RU486+) and without knockdown (RU486-) are statistically significant with * - $p < 0.05$ (Mann-Whitney U-test).

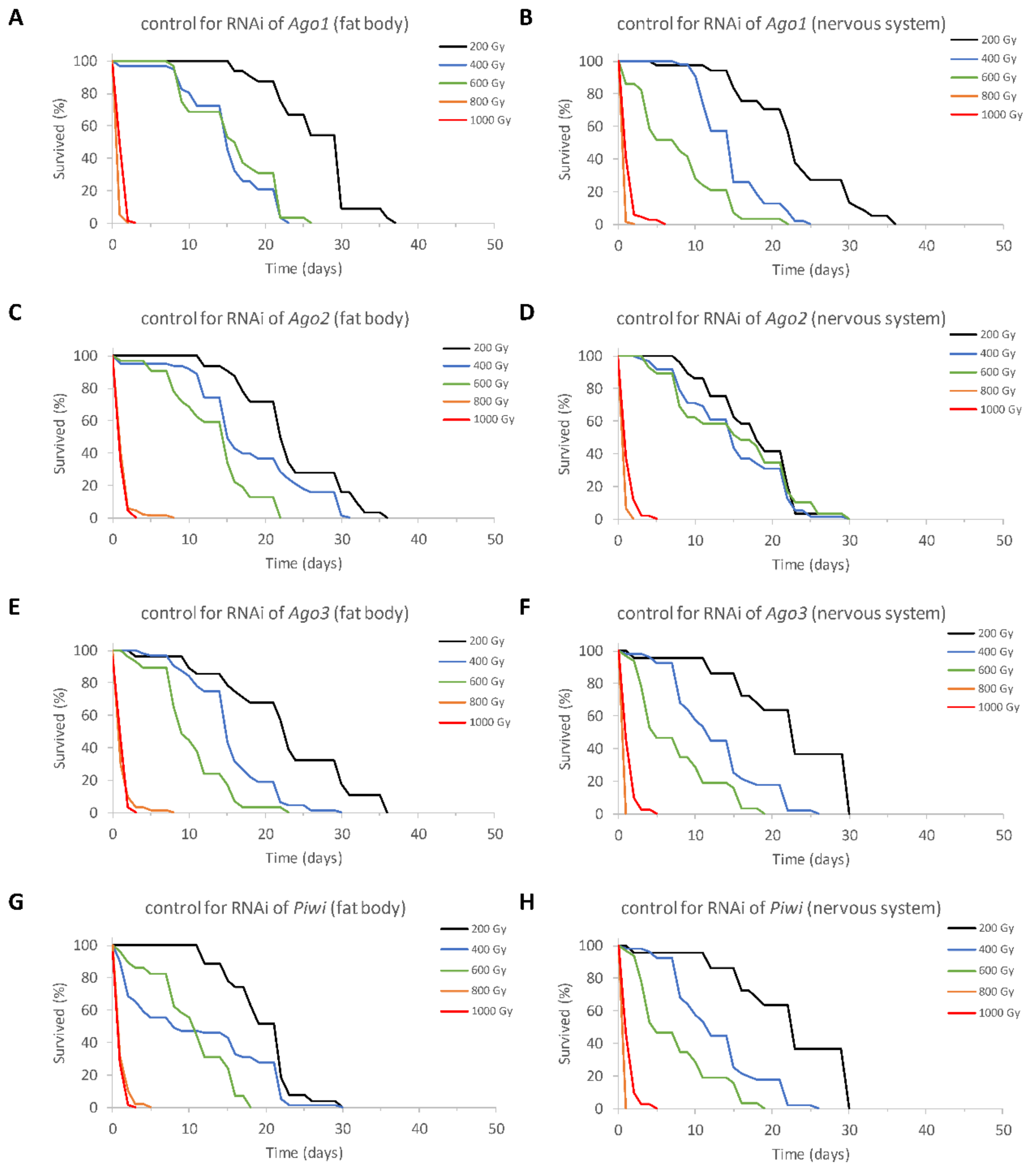

Figure S5. Effects of acute gamma irradiation on the survival of *Drosophila* male imago from the controls for RNAi of *AGO1* (**a, b**), *AGO2* (**c, d**), *AGO3* (**e, f**), *piwi* (**g, h**) in the fat body (**a, c, e, g**) and nervous system (**b, d, f, h**).

Table S10. Primers for real-time PCR.

Gene	Forward primer	Reverse primer
β-Tubulin	GCAACTCCACTGCCATCC	CCTGCTCCTCCTCGAACT
RpL32	GAAGCGCACCAAGCACTTCATC	CGCCATTTGTGCGACAGCTTAG
EF1α	AGGGCAAGAAGTAGCTGGTTTGC	GCTGCTACTACTGCGTGTTGTTG
AGO1	TGCGTCGCAAGTATCGTGTGTG	TCCAGCTGCAGTGGGAATGATTG
AGO2	ATCTACTACCGAGATGGCGTGAGC	TCTTGGGTTTACAGCCCACCTTG
AGO3	AACACTCGCATCTTCTCGGGTAGC	TCTACGACAGTTCCTGGCAATGGG
piwi	AATTCCTGAGCTCTGCCGAGTG	TCATGGCACGCATAAGCTGAAAG
HeT-A1	CGCGCGGAACCCATCTTCAGA	CGCCGCAGTCGTTTGGTGAGT
R1-element	CGTTGTTTCCACTGCCGTTA	CCTAGGCTGCGGAAACTGAT
Rt1a	CCACACAGACTGAGGCAGAA	ACGCATAACTTTCCGGTTTG
1731	AGCAAACGTCTGTTGGAAGG	CGACAGCAAAACAACACTGC
412	CACCGGTTTGGTCGAAAG	GGACATGCCTGGTATTTTGG
blood	TGCCACAGTACCTGATTTCG	GATTCGCCTTTTACGTTTGC
opus	CGAGGAGTGGGGAGAGATTG	TGCGAAAATCTGCCTGAACC
roo	CGTCTGCAATGTACTGGCTCT	CGGCACTCCACTAACTTCTCC
microsatellite/LINE-1	GGCCATGTCCGTCTGTCC	AGCTAGTGTGAATGCGAACG
Sod1	TGCACGAGTTCGGTGACAACAC	TCCTTGCCATACGGATTGAAGTGC
Prx5	CCGATGAGCTGAAGTCCAAG	TTGCCGTTCTCCACCACCAG
Gadd45	AAGTCGCGCACAGATACTCACG	TTTGTTGGTTCGGCAGCTGGTC
Xpc	AGAAGACGGTGCATTTGAGATTGC	ATGGGATGACAAGCGCCTTGATG
Ku80	AGCTTCAGAATGTCGCAACTACC	TCGTTGAAATCGAAGAGCAGGAG
spn-B	ATCACGCAATCCCATCGAGGAC	TCCGGTGCGAGAACATTAACCTG
Hsp27	ACTGGGTCGTCGTCGTTATTCG	CGCGCGACGTGACATTTGATTG
Hsp68	TGGGCACATTCGATCTCACTGG	TAACGTCGATCTTGGGCACTCC
Atg1	AGACTCTTCCTCGTGCAACTAGC	GCTTGAGATCACGATGCACAATTC
Atg5	CTCGTCAAGCTCAACTCCAAGG	GTTGACCAATCCCAGCCAAAGC
Ire1	GACAGTGAGGACAGCCGAATTATC	GCGATTGCGGATCCTTGTGTATC
